# MerTK-dependent efferocytosis by monocytic-MDSCs mediates resolution of ischemia/reperfusion injury after lung transplant

**DOI:** 10.1172/jci.insight.179876

**Published:** 2024-08-22

**Authors:** Victoria Leroy, Denny J. Manual Kollareth, Zhenxiao Tu, Jeff Arni C. Valisno, Makena Woolet-Stockton, Biplab Saha, Amir M. Emtiazjoo, Mindaugas Rackauskas, Lyle L. Moldawer, Philip A. Efron, Guoshuai Cai, Carl Atkinson, Gilbert R. Upchurch, Ashish K. Sharma

**Affiliations:** 1 Department of Surgery;; 2 Department of Pharmacology and Therapeutics;; 3 Department of Medicine, Division of Pulmonary, Critical Care and Sleep Medicine, College of Medicine, University of Florida, Gainesville, Florida, USA.; 4 Department of Biostatistics, College of Public Health and Health Professions and College of Medicine, University of Florida, Gainesville, Florida, USA.

**Keywords:** Immunology, Transplantation, Innate immunity, Monocytes, Surgery

## Abstract

Lung transplantation (LTx) outcomes are impeded by ischemia/reperfusion injury (IRI) and subsequent chronic lung allograft dysfunction (CLAD). We examined the undefined role of receptor Mer tyrosine kinase (MerTK) on monocytic myeloid-derived suppressor cells (M-MDSCs) in efferocytosis to facilitate resolution of lung IRI. Single-cell RNA sequencing of lung tissue and bronchoalveolar lavage (BAL) from patients after LTx were analyzed. Murine lung hilar ligation and allogeneic orthotopic LTx models of IRI were used with BALB/c (WT), *Cebpb*^–/–^ (MDSC-deficient), *Mertk^–/–^*, or MerTK–cleavage-resistant mice. A significant downregulation in MerTK-related efferocytosis genes in M-MDSC populations of patients with CLAD was observed compared with healthy individuals. In the murine IRI model, a significant increase in M-MDSCs, MerTK expression, and efferocytosis and attenuation of lung dysfunction was observed in WT mice during injury resolution that was absent in *Cebpb^–/–^* and *Mertk^–/–^* mice. Adoptive transfer of M-MDSCs in *Cebpb^–/–^* mice significantly attenuated lung dysfunction and inflammation. Additionally, in a murine orthotopic LTx model, increases in M-MDSCs were associated with resolution of lung IRI in the transplant recipients. In vitro studies demonstrated the ability of M-MDSCs to efferocytose apoptotic neutrophils in a MerTK-dependent manner. Our results suggest that MerTK-dependent efferocytosis by M-MDSCs can substantially contribute to the resolution of post-LTx IRI.

## Abstract 2

Effective clearance of dead cell debris by monocytic subset of myeloid derived suppressor cells via MerTK receptor signaling regulates post-lung transplant injury and graft dysfunction

## Graphical abstract

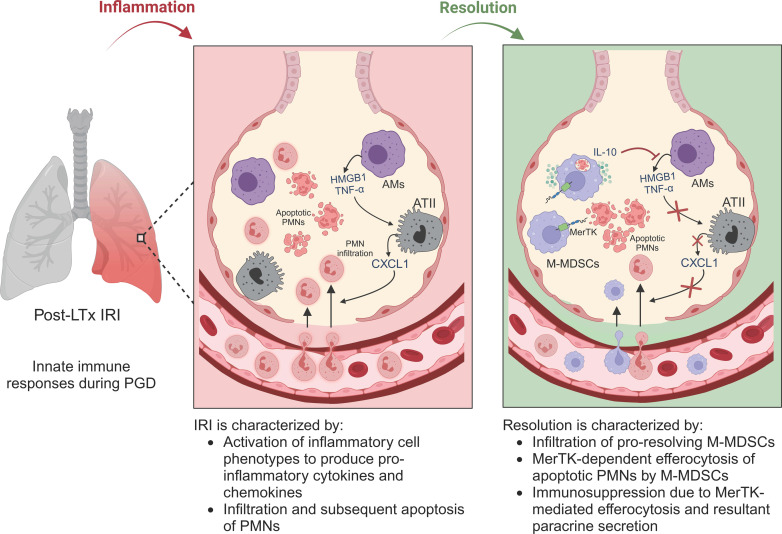

## Introduction

Lung transplantation (LTx) for patients with end-stage lung diseases is a lifesaving option that requires further investigations because of suboptimal clinical outcomes. As post-LTx ischemia/reperfusion injury (LTx-IRI) can lead to primary graft dysfunction (PGD) and chronic rejection (chronic lung allograft dysfunction; CLAD) causing the worst outcomes of all solid organ transplants, effective therapeutic modalities are urgently required to circumvent mortality in LTx recipients (1–3). IRI is characterized by immune cell infiltration and activation, increased vascular permeability, and production of inflammatory mediators, including reactive oxygen species (4). Dysregulation of endogenous mechanisms of inflammation resolution lead to exacerbated tissue injury and graft dysfunction (5, 6). Since PGD development is a primary risk factor for CLAD, it is imperative to understand endogenous mechanisms of inflammation resolution that can be harnessed to facilitate graft acceptance and tissue homeostasis.

Broadly, the inflammation resolution process is hallmarked by a variety of factors that include the presence and activation of immunosuppressive cells, the cessation of pro-inflammatory cell infiltration, and subsequent clearance of apoptotic cells (efferocytosis) to prevent secondary necrosis (7). Among immunosuppressive cell populations, myeloid-derived suppressor cells (MDSCs) have garnered research interest in the transplantation field both as a therapeutic measure as well as a diagnostic biomarker (8–10). This heterogenous cell population, comprising monocytic (M-) and granulocytic (G- or PMN-) MDSCs, are notable for their myriad immunosuppressive/pro-resolving actions, including modulation of cytokines, exhaustion of pro-inflammatory cells, and promotion of pro-resolving cell phenotypes (11–14). A pivotal characteristic of the resolution phase is efferocytosis, which involves the clearance of apoptotic cells and debris (15). This key pro-resolving action is carried out by both professional and nonprofessional phagocytes at the direction of various pro-efferocytic receptors, including Mer proto-oncogene tyrosine kinase (MerTK) (16, 17). Although the phagocytic role of macrophages is well characterized, the contribution to the efferocytic process by infiltrating monocytic subsets, such as M-MDSCs, in resolution of tissue inflammation remains to be deciphered. A recent study reported that infiltrating interstitial macrophages, but not alveolar macrophages, contribute primarily to efferocytosis of apoptotic alveolar type II epithelial cell apoptosis in lung inflammation following influenza (18). When efferocytosis is dysregulated, accumulation of apoptotic cell debris can exacerbate the inflammatory response and lead to tissue dysfunction (19). However, the role of efferocytosis and dysregulation of inflammation resolution pathways via MerTK and infiltrating monocytic subsets in post-LTx IRI remains to be delineated.

In this study, we investigated if M-MDSCs contribute to the resolution of lung IRI via MerTK-dependent efferocytosis leading to graft survival. Our results signify the importance of dysregulation of inflammation-resolution because of MerTK-cleavage and subsequent defective efferocytosis by M-MDSCs that contributes to exacerbated tissue inflammation and post-LTx PGD.

## Results

### Efferocytosis-related genes are downregulated in M-MDSCs in CLAD.

**Figure 1:**
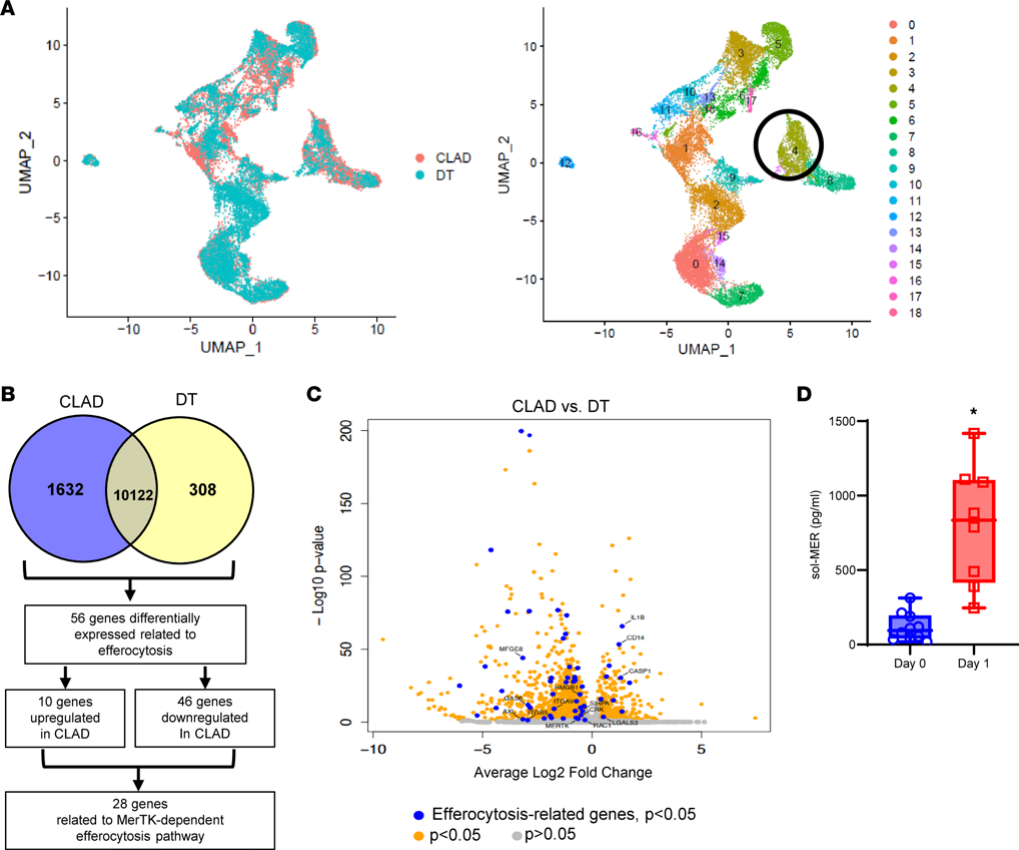
Single-cell RNA-sequencing analysis of myeloid cells reveals downregulation of efferocytosis-related genes in M-MDSCs of patients with CLAD. (**A**) Uniform manifold approximation and projection (UMAP) visualization of 18 myeloid cell clusters in lung tissue of patients with CLAD and donor controls. (**B**) Venn diagram outlining identification of DEGs for MerTK-dependent efferocytosis. Downregulated genes in CLAD (blue) and DT (yellow) are described. (**C**) Volcano plot of DEGs of myeloid cell cluster 4. Genes identified by blue dots are ERGs with differential expression of *P* < 0.05. Orange dots are other DEGs with *P* < 0.05. Gray dots are genes that are not significant (*P* > 0.05). (**D**) Quantification of sol-MER levels in BAL of patients showed significant increase on day 1 after LTx compared with day 0. Box plots show the interquartile range, median (line), and minimum and maximum (whiskers). Data analyzed by Mann-Whitney test; *P < 0.0001; *n* = 8–10/group.

We analyzed single-cell RNA-sequencing data from patients with CLAD and donor tissue (DT) from a recently reported study to identify differences in M-MDSC populations from myeloid cell–specific clusters (20). After data normalization and principal component analysis, we identified a total of 18 myeloid cell clusters using uniform manifold approximation and projection (UMAP) (Figure 1A). Subcluster analysis of these myeloid populations revealed cluster 4 as M-MDSCs based on expression of *HLA-DRA*, *ITGAM*, *CD33*, *CD14*, *FUT4*, *IL-10*, and *VEGFA*, as previously described (Supplemental Figure 1; supplemental material available online with this article; https://doi.org/10.1172/jci.insight.179876DS1) (21, 22). M-MDSCs, which display a variety of immunosuppressive capabilities, and therefore may contribute to graft tolerance, were present in patients with chronic rejection, which prompted further investigation.

Analysis of differentially expressed genes (DEGs) in M-MDSCs detected 1,632 genes downregulated in CLAD and 308 genes downregulated in DT (Supplemental Table 1). Since PGD can contribute to chronic rejection, we further explored differentially expressed efferocytosis-related genes (ERGs; Supplemental Table 2) in patients with CLAD, which is vital to inflammation resolution and especially crucial in pulmonary IRI (23). In-depth analysis revealed 5 of the top 30 DEGs (*CD163*, *C25H*, *PELI1*, *IL10*, *VSIG4*) were related to a downregulation in efferocytosis, and overall 56 DEGs were related to efferocytosis. Of the 56 ERGs, 46 were downregulated in CLAD (Supplemental Table 2), the majority of which were directly related to MerTK-mediated efferocytosis, which includes *MerTK*, *AXL*, *GAS6*, *ADAM9*, *SIRP*α, *CASP1*, and *RAC1* among others (Figure 1, B and C, and Supplemental Table 3). MerTK, as a cell surface receptor, is subject to cleavage, which contributes to defective efferocytosis (24). Since the cleavage of MerTK results in quantifiable soluble MER (sol-MER) in fluid secretions, we analyzed patient bronchoalveolar lavage (BAL) on days 0 and 1 after LTx. sol-MER was significantly increased in patients on day 1 after LTx (802.0 ± 142.7 vs. 117.5 ± 29.8 pg/mL; *P* < 0.0001) compared with day 0 (Figure 1D). These clinical findings prompted us to further explore the endogenous mechanisms associated with MerTK-dependent efferocytosis, via M-MDSCs, in our preclinical models of lung IRI.

### M-MDSCs mediate the endogenous resolution of experimental lung IRI.

**Figure 2:**
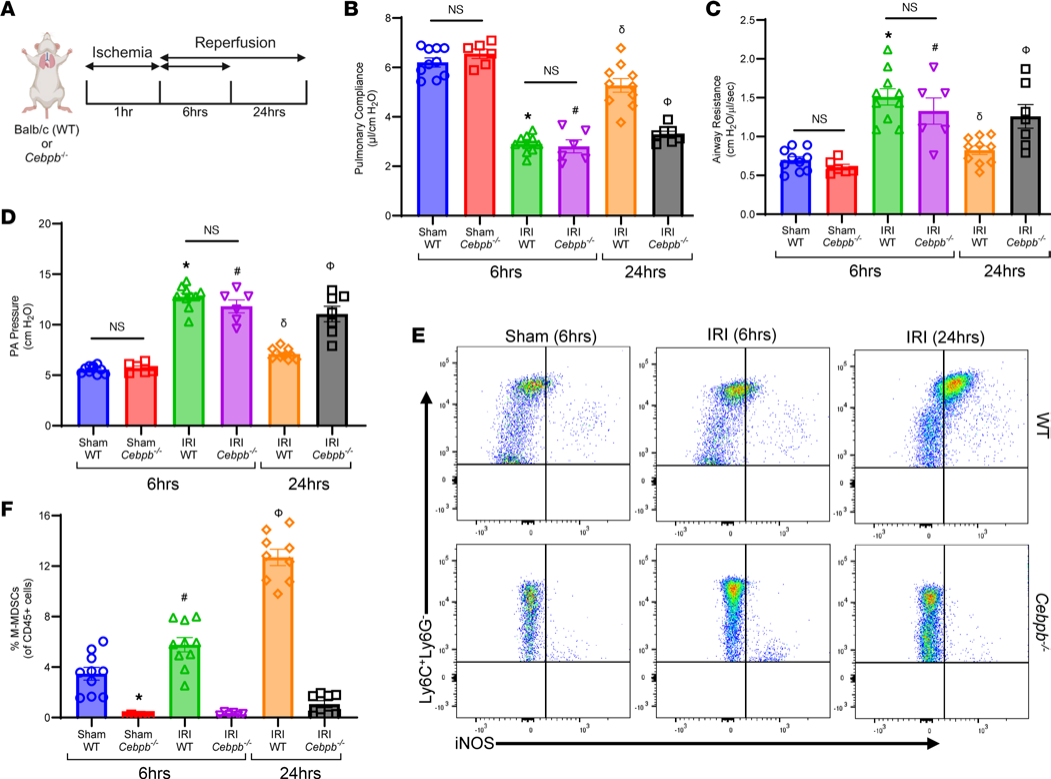
Increase in M-MDSCs is associated with the endogenous resolution of lung IRI. (**A**) Representative schematic depicting the hilar ligation IRI model where left lung undergoes 1 hour of ischemia followed by 6 or 24 hours of reperfusion in BALB/c (WT) and *Cebpb^–/–^* mice. (**B**–**D**) Significant lung dysfunction was observed in WT and *Cebpb^–/–^* mice following 6 hours compared with sham controls that was mitigated after 24 hours. However, lung dysfunction was significantly higher in *Cebpb^–/–^* mice compared with WT mice after 24 hours. *P < 0.0001 vs. WT sham; ^#^*P* < 0.0001 vs. *Cebpb^–/–^* sham; ^δ^*P* < 0.0001 vs. WT IRI (6 hours); ^Φ^*P* < 0.05 vs. WT IRI (24 hours); *n* = 6–10/group. (**E** and **F**) The percentage of M-MDSCs was significantly upregulated in WT mice following IRI (24 hours) compared with IRI (6 hours) or sham mice, as analyzed by flow cytometry. A markedly significant mitigation of M-MDSCs was observed in *Cebpb^–/–^* mice compared with WT mice. *P = 0.0005 vs. WT sham; ^#^*P* < 0.009 vs. WT sham and *Cebpb^–/–^* IRI (6 hours); ^δ^*P* < 0.0001 vs. WT IRI (6 hours) and *Cebpb^–/–^* IRI (24 hours); *n* = 6–10/group. Data analyzed by 1-way ANOVA and Tukey’s multiple comparisons test.

Using the murine hilar ligation model of lung IRI (Figure 2A), we observed that maximal lung dysfunction occurred at IRI (6 hours) compared with sham controls, as seen by decreased pulmonary compliance (2.9 ± 0.1 vs. 6.2 ± 0.2 μL/cmH_2_O; *P* < 0.0001) and increased airway resistance (1.5 ± 0.1 vs. 0.6 ± 0.04 cmH_2_O/μL/s; *P* < 0.0001) and pulmonary artery (PA) pressure (12.7 ± 0.4 vs. 5.6 ± 0.1 cmH_2_O; *P* < 0.0001). Furthermore, lung dysfunction was attenuated at IRI (24 hours) compared with IRI (6 hours), indicating endogenous resolution of lung dysfunction (Figure 2, B–D). Next, we investigated the contribution of M-MDSCs to inflammation resolution and lung injury attenuation using *Cebpb^–/–^* (MDSC-deficient) mice. Following IRI (6 hours), there was substantial lung dysfunction in *Cebpb^–/–^* mice compared with respective sham controls, as observed by significant decrease in pulmonary compliance (2.8 ± 0.3 vs. 6.5 ± 0.2 μL/cmH_2_O; *P* < 0.0001) as well as significant increase in airway resistance (1.3 ± 0.2 vs. 0.61 ± 0.04 cmH_2_O/μL/s; *P* < 0.0013) and PA pressure (11.8 ± 0.6 vs. 5.7 ± 0.2 cmH_2_O; *P* < 0.0001). There was no significant difference in lung dysfunction between WT and *Cebpb*^–/–^ mice during peak inflammation phase (Figure 2, B–D).

However, sustained lung dysfunction was observed in *Cebpb^–/–^* mice following IRI (24 hours), contrary to WT mice, which displayed endogenous resolution at 24 hours, as observed with significantly decreased pulmonary compliance (3.1 ± 0.1 vs. 5.2 ± 0.3 μL/cmH_2_O; *P* < 0.0001) and increased airway resistance (1.2 ± 0.2 vs. 0.8 ± 0.05 cmH_2_O/μL/s; *P* < 0.05) as well as PA pressure (11.2 ± 0.6 vs. 7.1 ± 0.2 cmH_2_O; *P* < 0.0001), indicating dysregulated resolution (Figure 2, B–D). There was no difference in lung function among WT and *Cebpb^–/–^* sham groups after 24 hours (data not shown).

Interestingly, WT mice displayed a significant increase in the M-MDSC population (CD45^+^CD11b^+^CD11c^–^Ly6G^–^Ly6C^+^iNOS^+^) infiltrating in the lungs during resolution phase (24 hours) compared with inflammation phase (6 hours) post-IRI (12.7 ± 0.6% vs. 5.7 ± 0.5%; *P* < 0.0001; Figure 2, E and F, and Supplemental Figure 2). There was a significant decrease in M-MDSCs in *Cebpb^–/–^* mice compared with WT mice after IRI (24 hours) (1 ± 0.3% vs. 12.7 ± 0.6%; *P* < 0.0001; Figure 2, E and F). On the contrary, G-MDSC (CD45^+^CD11b^+^CD11c^–^Ly6C^lo^Ly6G^+^) infiltration was significantly elevated following IRI (6 hours) compared with shams, but was decreased after IRI (24 hours), suggesting that G-MDSCs are associated with lung inflammation but not resolution of IRI (Supplemental Figure 3, A–C). These results highlight a critical association between M-MDSC infiltration and inflammation resolution.

**Figure 3:**
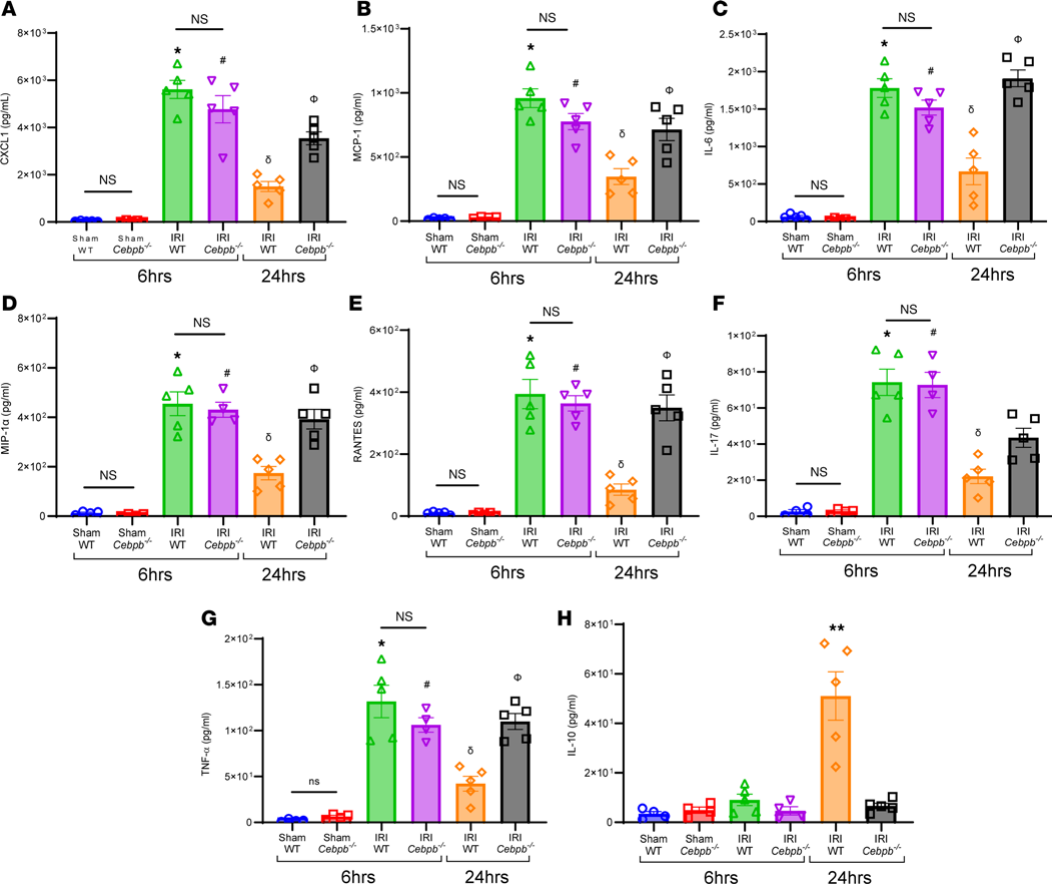
Pro-inflammatory cytokine expression was significantly increased in *Cebpb^–/–^* mice after IRI. (**A**–**G**) Pro-inflammatory cytokine and chemokine levels in BAL fluid were significantly increased in WT and *Cebpb^–/–^* mice after 6 hours of IRI compared with sham controls and were mitigated in WT mice following 24 hours but not in *Cebpb^–/–^* mice; *P < 0.0001 vs. WT sham; ^#^*P* < 0.0001 vs. *Cebpb^–/–^* sham; ^δ^*P* < 0.0001 vs. WT IRI (6 hours); ^Φ^*P* < 0.003 vs. WT IRI (24 hours) *n* = 4–5/group. (**H**) Antiinflammatory IL-10 expression was significantly increased in WT mice following 24 hours compared with all other groups. **P < 0.0001 vs. other groups; *n* = 4–5/group. Data analyzed by 1-way ANOVA and Tukey’s multiple comparisons test.

**Figure 4:**
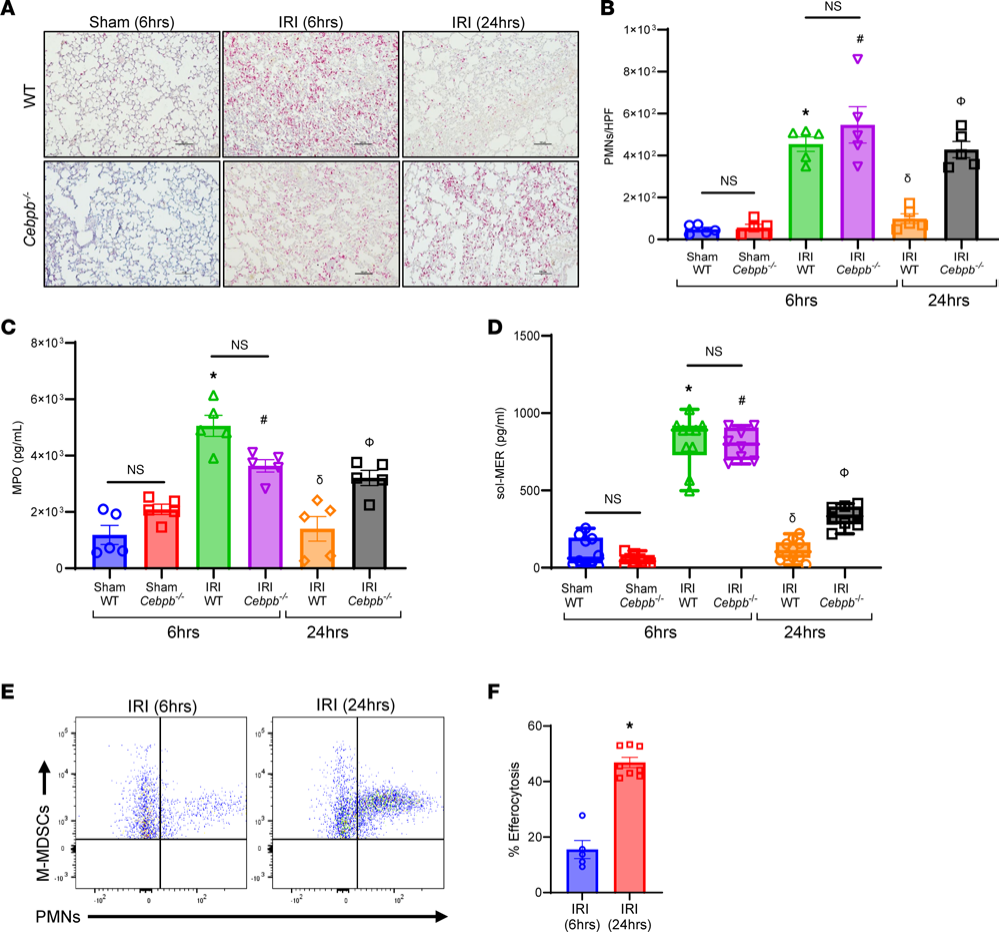
*Cebpb^–/–^* mice experienced sustained neutrophil infiltration and activation following IRI. (**A**–**C**) PMN infiltration in lung tissue and activation (MPO levels in BAL) were significantly increased in WT and *Cebpb^–/–^* mice after 6 hours compared with sham controls, which were attenuated in WT mice following 24 hours but not in *Cebpb^–/–^* mice. *P < 0.0001 vs. WT sham; ^#^*P* < 0.02 vs. *Cebpb^–/–^* sham; ^δ^*P* < 0.0001 vs. WT IRI (6 hours); ^Φ^*P* < 0.01 vs. WT IRI (24 hours); *n* = 5/group. PMNs were quantified per high-power field (HPF). Scale bars represent 100 μm. (**D**) sol-MER levels in BAL were significantly increased in both WT and *Cebpb^–/–^* mice after 6 hours compared with respective sham controls. These levels were mitigated in WT mice and significantly decreased in *Cebpb^–/–^* mice after IRI (24 hours) compared with IRI (6 hours). Box plots show the interquartile range, median (line), and minimum and maximum (whiskers). *P < 0.0001 vs. WT sham; ^#^*P* < 0.0001 vs. *Cebpb^–/–^* sham; ^δ^*P* < 0.0001 vs. WT IRI (6 hours); ^Φ^*P* < 0.0004 vs. WT IRI (6 hours and 24 hours) and *Cebpb^–/–^* IRI (6 hours); *n* = 8–10/group. Data analyzed by 1-way ANOVA and Tukey’s multiple comparisons test. (**E** and **F**) Efferocytosis of neutrophils by M-MDSCs was significantly increased after 24 hours compared with 6 hours in WT mice following IRI. *P = 0.0016 vs. IRI (6 hours); *n* = 5–8/group. Data analyzed by Mann-Whitney test.

The lung dysfunction observed after IRI (6 hours) in WT and *Cebpb^–/–^* mice was accompanied by an increase in proinflammatory cytokines (CXCL1, MCP-1, IL-6, MIP-1α, RANTES, IL-17, and TNF-α) as well as a decrease in antiinflammatory IL-10 secretion in BAL (Figure 3, A–H). Polymorphonuclear neutrophil (PMN) infiltration and activation (measured by myeloperoxidase; MPO) were elevated in both WT and *Cebpb^–/–^* at IRI (6 hours) compared with respective sham controls (Figure 4, A–C). PMN infiltration as well as activation were significantly increased in *Cebpb^–/–^* mice compared with WT mice at 24 hours after IRI (428.4 ± 38.5 vs. 99 ± 23.1 PMNs/high-power field (HPF); *P* = 0.0002; and 3,207 ± 269.6 vs. 1,400 ± 438.5 pg/mL; *P* = 0.005, respectively), indicating dysregulation of PMN efferocytosis in *Cebpb^–/–^* mice (Figure 4, A–C).

To further explain the differences in PMN infiltration and activation, we investigated the processes that regulate the endogenous clearance of these cells. Both WT and *Cebpb^–/–^* demonstrated increased sol-MER levels compared with respective sham controls after IRI. sol-MER levels were significantly decreased in WT mice during resolution following IRI (24 hours) compared with the inflammation phase of IRI (6 hours) (139.9 ± 24.6 vs. 796.2 ± 73.5 pg/mL; *P* < 0.0001; Figure 4D). However, *Cebpb^–/–^* mice displayed significantly elevated sol-MER levels compared with WT mice at 24 hours (331.6 ± 24 vs. 113.4 ± 20.7 pg/mL; *P* = 0.0004; Figure 4D). Moreover, mRNA expression of *MerTK* in lung tissue was significantly increased following 24 hours in WT mice compared with 6 hours (Supplemental Figure 4). Importantly, the endogenous efferocytosis by M-MDSCs in WT mice was significantly upregulated following IRI (24 hours) compared with IRI (6 hours) (46.9 ± 1.9% vs. 15.5 ± 3.2%; *P* = 0.0016; Figure 4, E and F). Taken together, these data suggest that M-MDSCs are critical to the inherent resolution of lung IRI and mitigate inflammatory cytokine secretion, leukocyte trafficking, and efferocytosis.

### Adoptive transfer of M-MDSCs attenuates pulmonary dysfunction after lung IRI.

**Figure 5:**
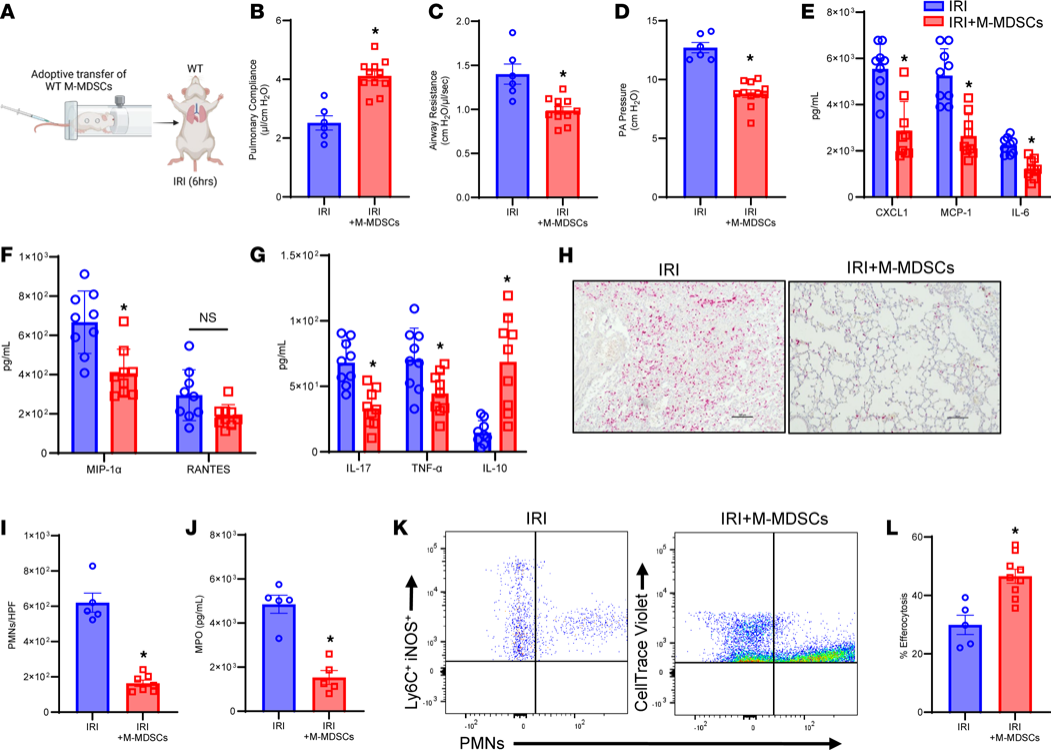
Administration of M-MDSCs attenuates pulmonary dysfunction after IRI. (**A**) Schematic depicting adoptive transfer of WT M-MDSCs prior to IRI (6 hours) in WT mice. (**B**–**D**) Treatment with M-MDSCs significantly improved lung dysfunction compared with untreated mice. *P < 0.003 vs. IRI; *n* = 6–11/group. (**E**–**G**) Pro-inflammatory cytokines in BAL fluid were significantly reduced, and antiinflammatory IL-10 expression was significantly increased in M-MDSC–treated mice compared with untreated mice. *P < 0.05 vs. IRI; *n* = 9/group. (**H**) Representative images of histological staining for PMNs after IRI with and without M-MDSC treatment. Scale bars represent 100 μm. (**I** and **J**) Neutrophil infiltration in lung tissue and MPO levels in BAL were significantly attenuated in mice treated with M-MDSCs. *P < 0.008 vs. IRI; *n* = 5/group. (**K** and **L**) Adoptively transferred M-MDSCs (CellTrace Violet) demonstrated a significant increase in efferocytosis of apoptotic neutrophils compared with endogenous M-MDSCs (Ly6C^+^iNOS^+^) in lung tissue. *P = 0.004 vs. IRI; *n* = 5–9/group. Data analyzed by Mann-Whitney test.

To elucidate the reparative role of M-MDSCs in inflammation resolution, we performed adoptive transfer of exogenous M-MDSCs (generated in vitro; Supplemental Figure 5) (25) in WT mice before IRI (6 hours) (Figure 5A). WT mice treated with M-MDSCs showed significant improvement in lung function compared with untreated mice after IRI (6 hours), as demonstrated by an increase in pulmonary compliance (4.1 ± 0.2 vs. 2.5 ± 0.2 μL/cmH_2_O; *P* < 0.001) as well as decrease in airway resistance (1.0 ± 0.04 vs. 1.4 ± 0.1 cmH_2_O/μL/s; *P* < 0.001) and PA pressure (8.8 ± 0.5 vs. 12.7 ± 0.4 cmH_2_O; *P* < 0.003) (Figure 5, B–D). Additionally, M-MDSC–treated mice had significantly decreased proinflammatory cytokines and increased antiinflammatory/pro-resolving cytokine, IL-10, compared with untreated mice after IRI (6 hours) (Figure 5, E–G). The secretion of high mobility group box 1 in BAL, primarily secreted by alveolar macrophages following IRI (26), was significantly mitigated following M-MDSC treatment in WT mice compared with untreated mice after IRI (6 hours) (80 ± 12.0 vs. 154.9 ± 21.3 ng/mL; *P* = 0.01; Supplemental Figure 6A). Moreover, M-MDSC–treated mice had significantly decreased neutrophil infiltration (163.7 ± 18.2 vs. 620.0 ± 54.3; PMNs/HPF; *P* = 0.0025) (Figure 5, H and I) and MPO levels (1,533.0 ± 318.0 vs. 4,851.0 ± 410.0 pg/mL; *P* = 0.0079) (Figure 5J) compared with untreated controls, suggesting increased efferocytosis of neutrophils by exogenously administered M-MDSCs. Adoptively transferred M-MDSCs also significantly increased efferocytosis of exogenously administered apoptotic PMNs compared with endogenous M-MDSCs after IRI (6 hours) (46.5 ± 2.4% vs. 29.9 ± 3.2%; *P* = 0.004) (Figure 5, K and L). In contrast, adoptive transfer of G-MDSCs did not offer protection against lung dysfunction after IRI compared with untreated mice (Supplemental Figure 3, D–F). These findings suggest that exogenously administered M-MDSCs are capable of mitigating pulmonary IRI.

### MerTK is critical to the endogenous resolution of lung IRI.

To understand the role of MerTK during efferocytosis and inflammation resolution, we analyzed *Mertk^–/–^* mice using the lung IRI model. *Mertk^–/–^* mice subjected to IRI (24 hours) failed to resolve, with a significant increase in lung dysfunction compared with C57BL/6 mice, as demonstrated by decreased pulmonary compliance (2.7 ± 1.7 vs. 5.4 ± 0.2 μL/cmH_2_O; *P* < 0.0001), as well as increased airway resistance (1.5 ± 0.1 vs. 0.7 ± 0.1 cmH_2_O/μL/s; *P* < 0.0001) and PA pressure (10.7 ± 0.4 vs. 6.3 ± 0.3 cmH_2_O; *P* < 0.0001; Supplemental Figure 7). No significant differences were observed in lung infiltration of M-MDSCs in *Mertk^–/–^* and C57BL/6 mice following IRI (24 hours) (17.3 ± 1.8% vs. 13.0 ± 1.2%; Supplemental Figure 8). Lung dysfunction was accompanied by a significant increase in proinflammatory cytokine and chemokine expression and significant decrease in IL-10 in BAL fluid from these mice compared with C57BL/6 mice (24 hours) (Supplemental Figure 9). Moreover, the *Mertk^–/–^* mice displayed significantly increased PMN infiltration (506.8 ± 30.9 vs. 54.8 ± 17.4 PMNs/HPF; *P* < 0.0001; Supplemental Figure 10, A and B) and MPO expression (5,321 ± 469.2 vs. 2,320 ± 668.9 pg/mL; *P* = 0.01; Supplemental Figure 10C) compared with C57BL/6 after IRI (24 hours), indicating dysregulated efferocytosis of PMNs in mice that lack MerTK (loss of function).

Conversely, we sought to examine the role of MerTK by assessing injury resolution in MerTK–cleavage-resistant (MerTK-CR; gain-of-function) mice (27). MerTK-CR mice were significantly protected after IRI (6 hours) compared with C57BL/6 mice, as seen by increased pulmonary compliance (3.6 ± 0.2 vs. 2.3 ± 0.2 μL/cmH_2_O; *P* = 0.007) as well as decreased airway resistance (1.0 ± 0.1 vs. 1.7 ± 0.03 cmH_2_O/μL/s; *P* < 0.0001) and PA pressure (8.8 ± 0.4 vs. 13.6 ± 0.4 cmH_2_O; *P* < 0.0001; Supplemental Figure 11). MerTK-CR mice demonstrated significant protection against lung inflammation, as observed by a decrease in proinflammatory cytokines and increase in antiinflammatory IL-10 expression compared with C57BL/6 mice after IRI (6 hours) (Supplemental Figure 12). Additionally, MerTK-CR mice displayed a significant decrease in PMN infiltration (148.9 ± 13.14 vs. 680.4 ± 44.24 PMNs/HPF; *P* < 0.0001; Supplemental Figure 13, A and B) and MPO expression (1,962 ± 291.5 vs. 4,591 ± 660.2 pg/mL; *P* = 0.0006; Supplemental Figure 13C) compared with C57BL/6 mice. The expression of MerTK was absent in *Mertk^–/–^* mice and detectable in MerTK-CR mice compared with C57BL/6 mice (Supplemental Figure 14, A and B). Taken together, these results demonstrate the critical role of MerTK in efferocytosis-mediated resolution of lung IRI.

### MerTK-mediated efferocytosis by M-MDSCs contributes to inflammation resolution during lung IRI.

**Figure 6:**
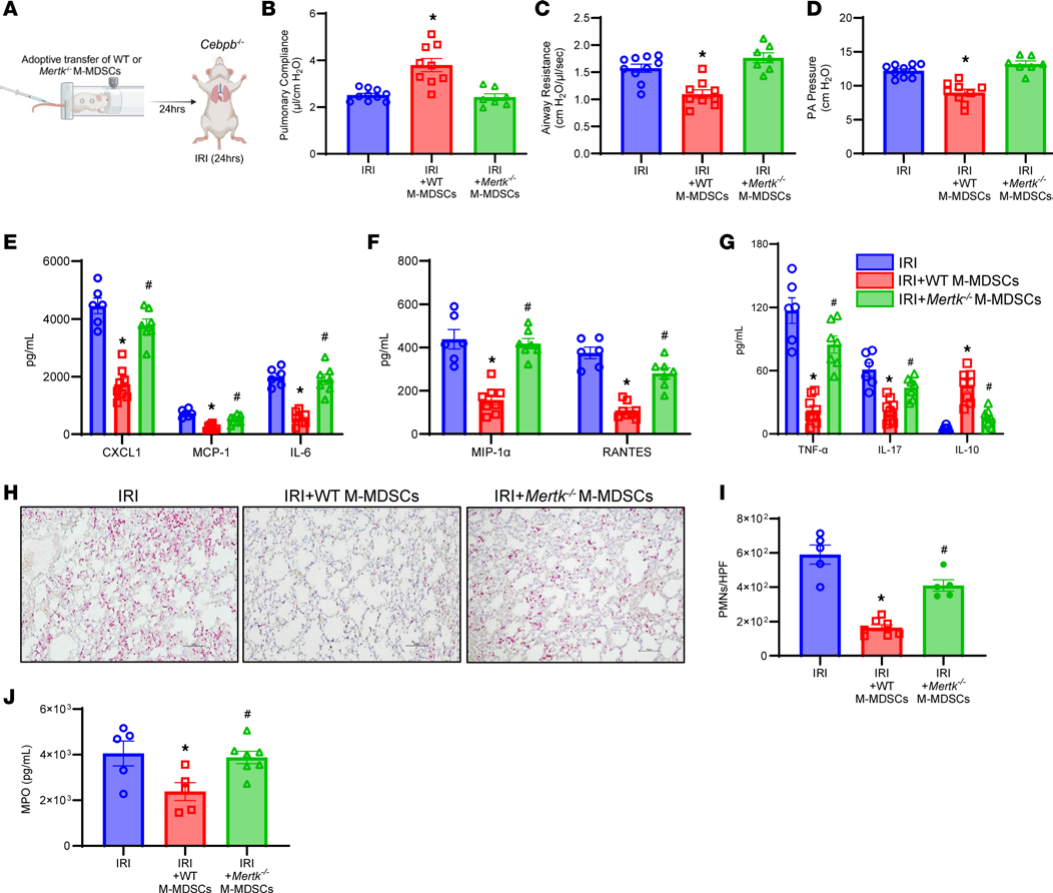
M-MDSC–mediated resolution of lung IRI is mediated via MerTK. (**A**) Schematic depicting adoptive transfer of M-MDSCs prior to IRI (24 hours) in *Cebpb^–/–^* mice. (**B**–**D**) Treatment with M-MDSCs from WT mice, but not from *Mertk*^–/–^ mice, significantly mitigated lung dysfunction compared with untreated mice. *P < 0.006 vs. other groups; *n* = 7–10/group. (**E**–**G**) Expression of pro-inflammatory cytokines was significantly decreased in mice treated with WT M-MDSCs but not with *Mertk^–/–^* M-MDSCs. *P < 0.05 vs. IRI; ^#^*P* < 0.05 vs. IRI+WT M-MDSCs; *n* = 6–8/group. (**H** and **I**) PMN infiltration was significantly attenuated by treatment with WT-derived M-MDSCs but not *Mertk^–/–^*-derived M-MDSCs. *P < 0.0001 vs. IRI; ^#^*P* = 0.0005 vs. IRI+WT M-MDSCs; *n* = 5–7/group. Scale bars represent 100 μm. (**J**) MPO levels in BAL were significantly mitigated by WT M-MDSCs but not by *Mertk^–/–^* M-MDSCs. *P < 0.05 vs. IRI alone; ^#^*P* < 0.05 vs. IRI + WT M-MDSCs; *n* = 5–7/group. Data analyzed by 1-way ANOVA and Tukey’s multiple comparisons test.

Next, we sought to understand the involvement of MerTK on M-MDSCs in the resolution of lung IRI. We performed adoptive transfer of WT and *Mertk^–/–^* M-MDSCs into *Cebpb^–/–^* before IRI (24 hours) (Figure 6A). *Cebpb^–/–^* mice treated with WT M-MDSCs demonstrated significant protection in lung dysfunction following IRI (24 hours) that was absent in untreated mice as observed by increased pulmonary compliance (3.8 ± 0.3 vs. 2.5 ± 0.1 μL/cmH_2_O) as well as decreased airway resistance (1.1 ± 0.1 vs. 1.6 ± 0.1 cmH_2_O/μL/s) and PA pressure (9.0 ± 0.5 vs. 12.2 ± 0.3 cmH_2_O). However, *Cebpb*^–/–^ mice treated with *Mertk^–/–^* M-MDSCs showed significantly increased lung dysfunction and injury compared with mice treated with WT M-MDSCs and were equivalent to untreated counterparts (Figure 6, B–D). Additionally, pro-inflammatory cytokines and chemokines were significantly mitigated in mice treated with WT M-MDSCs but not with *Mertk^–/–^* M-MDSCs (Figure 6, E–G). PMN infiltration following IRI (24 hours) (163.7 ± 18.2 vs. 409.4 ± 33.1 PMNs/HPF; *P* = 0.0005), as well as MPO expression (2,380.0 ± 393.7 vs. 3,873 ± 273.2 pg/mL; *P* = 0.04), were mitigated in mice treated with WT M-MDSCs compared with *Mertk^–/–^* M-MDSC–treated mice (Figure 6, H–J). No significant differences were observed in the trafficking of exogenously administered WT and *Mertk^–/–^* M-MDSCs to the lung tissue in *Cebpb^–/–^* mice following IRI (24 hours) (31.8 ± 0.9% vs. 35.9 ± 1.4%; Supplemental Figure 15).

### Apoptotic PMNs undergo efferocytosis by M-MDSCs in a MerTK-dependent manner.

**Figure 7:**
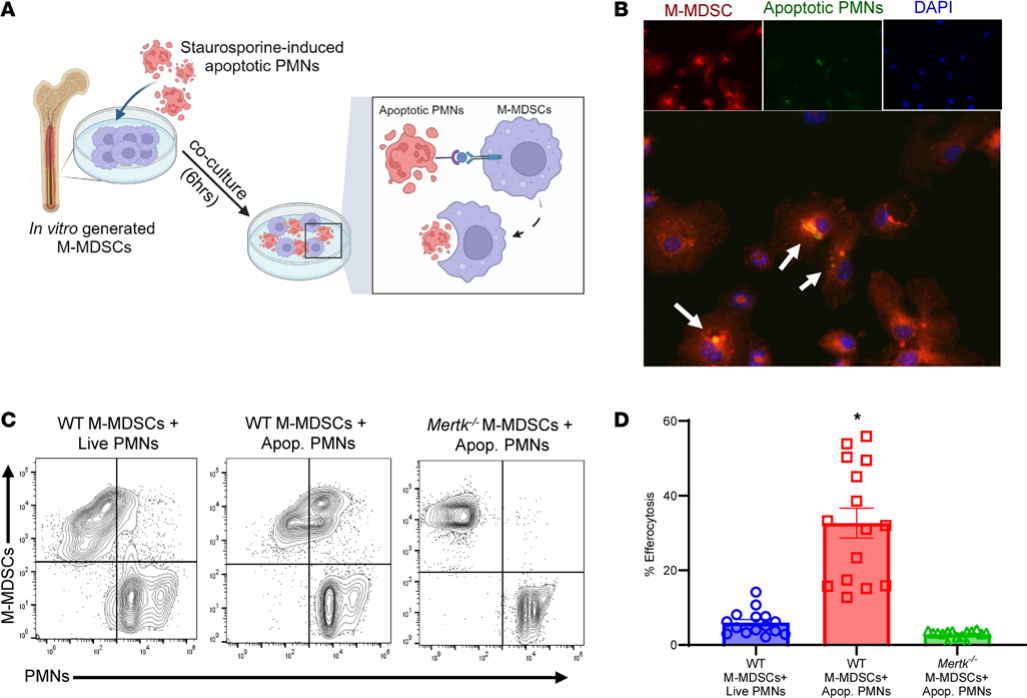
MerTK mediates M-MDSC–dependent efferocytosis in vitro. (**A**) Schematic depicting MerTK-mediated efferocytosis of apoptotic PMNs by M-MDSCs. (**B**) Representative immunofluorescence images demonstrating colocalization (shown by arrows) of M-MDSCs’ (red) engulfment of apoptotic PMNs (green). DAPI is shown in blue. Original magnification, 40×, representative images are shown. (**C** and **D**) Representative flow cytometry plots showing engulfment of apoptotic PMNs by WT or *Mertk^–/–^*-derived M-MDSCs. Quantification in lung tissue showed a significant increase in efferocytosis of apoptotic PMNs by WT-derived M-MDSCs but was absent in *Mertk^–/–^*-derived M-MDSCs. *P < 0.0001 vs. other groups; *n* = 15/group. Data analyzed by 1-way ANOVA and Tukey’s multiple comparisons test.

The integral role of MerTK-dependent efferocytosis by M-MDSCs was further investigated by our in vitro studies (Figure 7A). Confocal analysis of M-MDSCs cocultured with apoptotic PMNs demonstrated coexpression of ingested PMNs by MDSCs, signifying a marked increase in efferocytosis (Figure 7B). Quantitative analysis was performed using flow cytometry, which demonstrated a significant increase in uptake of apoptotic PMNs by WT M-MDSCs compared with *Mertk^–/–^* M-MDSCs (32.7 ± 4.0% vs. 2.9 ± 0.3%; *P* < 0.0001) (Figure 7, C and D). Furthermore, the immunosuppressive ability of M-MDSCs was evaluated by coculture experiments using invariant NKT cells that are known to preferentially secrete IL-17 during lung IRI (28). The expression of IL-17 in culture supernatants was significantly increased following hypoxia/reoxygenation (compared with normoxia controls), which was abrogated upon coculture with M-MDSCs (77.0 ± 10.8 vs. 39.4 ± 7.2 pg/mL; *P* = 0.001) (Supplemental Figure 6B). These results demonstrate the ability of M-MDSCs to mediate activation of key immune cells integral to lung IRI.

### Resolution of lung IRI in a murine orthotopic LTx model is mediated by M-MDSCs.

**Figure 8:**
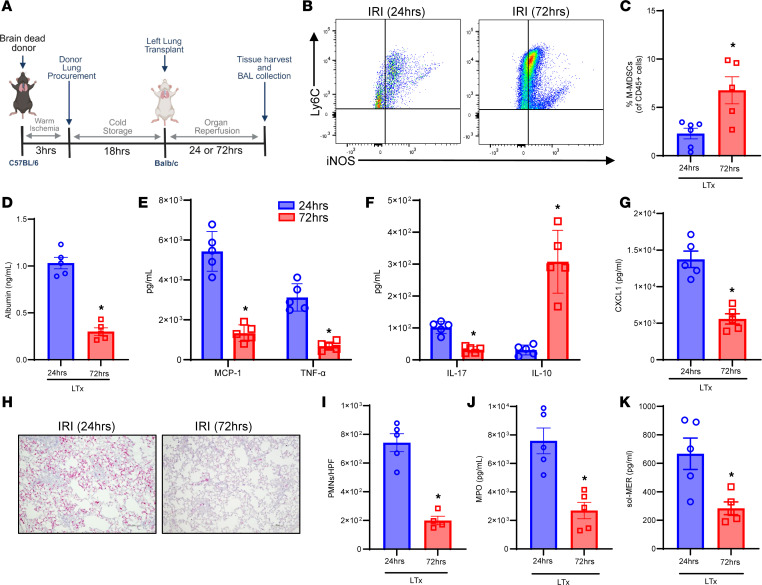
Resolution of IRI in an allogeneic orthotopic brain-dead model of IRI after LTx is associated with increase in M-MDSCs. (**A**) Schematic depicting brain-dead model of LTx in C57BL/6 donors and WT (BALB/c) recipients. (**B** and **C**) Representative flow cytometry plots and quantification of M-MDSCs in lung tissue from LTx recipients. The percentage of M-MDSCs was significantly upregulated after 72 hours of reperfusion to 24 hours. *P = 0.03 vs. 24 hours; *n* = 5–6/group. (**D**) Albumin levels in BAL were significantly mitigated following 72 hours of reperfusion compared with 24 hours of reperfusion. *P = 0.0079; *n* = 5/group. (**E**–**G**) Expression of pro-inflammatory cytokines in BAL was significantly mitigated and IL-10 expression was significantly increased after 72 hours compared with 24 hours of reperfusion. *P < 0.04; *n* = 5/group. (**H** and **I**) PMN infiltration in lung tissue was significantly abrogated following 72 hours of reperfusion compared with 24 hours. *P = 0.01; *n* = 4–5/group. Scale bars represent 100 μm. (**J**) MPO expression in BAL was significantly mitigated following 72 hours of reperfusion. *P = 0.0079; *n* = 5/group. (**K**) sol-MER levels in BAL were significantly decreased in LTx recipient mice following 72 hours of reperfusion compared with 24 hours. *P = 0.016; *n* = 5/group. Data analyzed by Mann-Whitney test.

To further validate the findings of the hilar ligation IRI model, we assessed the role of MerTK-dependent resolution of IRI in a clinically relevant murine orthotopic allogeneic LTx model (Figure 8A). Following 72 hours of after LTx reperfusion, there was a significant increase in M-MDSC infiltration compared with 24 hours of LTx IRI (7.7 ± 1.2% vs. 2.7 ± 0.4%; *P* = 0.016) (Figure 8, B and C). This increase in M-MDSCs at 72 hours coincided with a significant decrease in albumin levels (0.3 ± 0.04 vs. 1.0 ± 0.06 ng/mL; *P* = 0.007; Figure 8D) signifying decrease in lung edema, as well as marked decrease in proinflammatory cytokines and increase in IL-10 expression compared with post-LTx IRI at 24 hours (Figure 8, E–G). Furthermore, enhanced efferocytosis during resolution was accompanied by a significant decrease in PMN infiltration (216.7 ± 34.5 vs. 765.9 ± 49.7 PMNs/HPF; *P* = 0.0014; Figure 8, H and I), MPO expression (2,301 ± 929.5 vs. 8,537 ± 1,131 pg/mL; *P* = 0.014; Figure 8J), and sol-MER levels (283.9 ± 44.6 vs. 667.0 ± 110.6 pg/mL; *P* = 0.016; Figure 8K) compared with 24 hours after LTx IRI. Taken together, the results in both IRI models as well as in vitro studies underscore the pivotal efferocytic role of infiltrating M-MDSCs in lung tissue that immunomodulates the resolution of post-LTx IRI.

## Discussion

This study characterizes the importance of dysregulated resolution in PGD development, which remains a significant clinical burden for LTx patients as evidenced by the 6.2-year median survival rate (29, 30). The results reported herein describe a role of M-MDSCs in the resolution of lung IRI via efferocytosis of the apoptotic cell debris following post-LTx IRI. Using human LTx patient samples and 2 established models of lung IRI, our data delineate the mechanism of dysregulated clearance of dead cell debris that triggers uncontrolled inflammation and tissue injury and demonstrate this process as a crucial regulator for effective resolution of lung injury and graft survival. We identified an important role of the monocytic cell subset population that can regulate efferocytosis in 2 murine LTx models as well as in human LTx samples. The importance and clinical relevance of these studies are indicated by the pivotal role of MerTK receptors in mediating efferocytosis, suggesting that prevention of MerTK cleavage on monocytic immune cell populations can alleviate post-LTx IRI.

The resolution of inflammation caused by IRI, or any sterile insult, is a highly coordinated and intricate process. Reparative mechanisms for homeostatic conditions include cessation of pro-inflammatory leukocyte infiltration, upregulation of efferocytosis and enhanced production of pro-resolving molecules (i.e., antiinflammatory cytokines, specialized pro-resolving lipid mediators, etc.), as well as increase in pro-resolving/antiinflammatory cell population phenotypes (6, 7). A particularly severe insult can disrupt one or more of these interdependent processes, ultimately resulting in failed resolution. One notable pro-resolving cell population of the inflammatory response is the immunosuppressive M-MDSC subset that has pivotal immunoregulatory capabilities, and thus, can serve as a therapeutic modality in various disease conditions (31). However, the contribution of M-MDSCs in the resolution of post-LTx IRI remains to be deciphered. A recent report suggests that M-MDSC subset frequencies increase in peripheral blood after acute cellular rejection in LTx patients that could be influenced by immunosuppressive therapies (32). These findings underscore the importance of the complexity between immunosuppressive therapies, such as calcineurin or mTOR inhibition as well as dexamethasone administration in transplant patients, and endogenous immune regulation by monocytic populations, such as M-MDSCs. Since the functional capabilities of inherent monocytes may be affected because of cleavage of pro-efferocytic receptors, such as TAM (Tyro3, Axl, MerTK), secondary to inflammatory milieu or immunosuppressive therapies, our results suggest the importance of using alternate modalities to enhance immune regulation. While research surrounding the role of MDSCs in lung IRI is largely unexplored, previous studies in other organ transplant models have investigated their potential contribution (33, 34). A study of renal IRI found that depletion of both MDSC subsets via GR-1 antibody led to injury improvement and that adoptive transfer of both G- and M-MDSC subsets led to worsening of renal IRI (35). Instead of using strategies that deplete or adoptively transfer all MDSC subsets, we focused on specifically using the enriched MDSC subsets for our studies to delineate their specific contribution in lung IRI. We observed that adoptive transfer of only M-MDSCs provided resolution, whereas G-MDSCs failed to provide protection in the context of lung IRI.

The immunological response during lung IRI is notably characterized by the infiltration of PMNs, which eventually undergo cell death, such as apoptosis or NETosis, after the initial insult has subsided (23). The clearance, or efferocytosis, of these apoptotic cells is crucial for mitigating a feed-forward loop of continuing inflammation and tissue injury (15). Though this process is under regulation by a variety of receptors, MerTK is an effective efferocytic receptor because of its high level of expression in multiple tissues of the body as well as on primary phagocytes like macrophages (36). MerTK expressed on the cell surface is subject to proteolytic cleavage by ADAM17, among other molecules, resulting in the generation of sol-Mer (37). This decreases cell surface function of MerTK, but sol-Mer also serves as a soluble ligand that further decreases ligand-dependent interactions and activation of MerTK receptor (24). Impaired MerTK function can lead to worsening of inflammation and dysregulated repair mechanisms in atherosclerosis, bacteria-induced lung injury, and myocardial IRI, whereas prevention of MerTK cleavage with genetic or pharmacological techniques has been demonstrated to enhance inflammation resolution (38–40). Importantly, our study demonstrates the pivotal importance of preventing MerTK cleavage as a therapeutic strategy for mitigating lung injury and enhancing resolution. Moreover, since the levels of sol-Mer in BAL can act as a biomarker of the efferocytic process in the lung, an important indicator highlighted by our human patient and reciprocal murine studies, the relative contribution of MerTK-dependent efferocytosis can be potentially correlated with PGD and clinical outcomes.

In the quest for identifying therapeutic targets that can prevent MerTK cleavage, recent studies have elucidated the role of specialized pro-resolving lipid mediators, such as RvD1, to prevent MerTK cleavage on macrophages and enhance efferocytosis (27, 41). It is important to note that various cell types are capable of performing MerTK-dependent or -independent efferocytosis, and the relative contribution of each cell is highly dependent on the injured microenvironment. Alveolar macrophages are the primary tissue-resident professional phagocyte tasked with constant surveillance. Our previous studies have also demonstrated impaired efferocytosis of alveolar macrophages during peak inflammation (42). This is likely due to MerTK cleavage on alveolar macrophages during initial insult, whereas immune trafficking subsets, such as M-MDSCs, infiltrate the site of injury with functioning MerTK and thus effectively propagate efferocytosis to facilitate resolution. Thus, the sequential contribution and role of MerTK cleavage of alveolar macrophages during early inflammation and the subsequent swarming of infiltrating monocytes like M-MDSCs during the resolution phase likely play a crucial role in determining the fate of the lung allograft. Additionally, it is plausible that M-MDSCs are acting in a multifaceted manner not only for efferocytosis but also through secretion of paracrine factors (i.e., cytokines and extracellular vesicles), which remains to be further elucidated. Beyond efferocytosis, maintaining or enhancing MerTK function during acute injury has the potential to enhance antiinflammatory mediator production, increase antiinflammatory macrophage function, and regulate macrophage-dependent lipid metabolism (27, 43–45).

M-MDSCs are readily recruited to sites of inflammation through canonical monocyte trafficking pathways like the CCL2/CCR2 axis (46). Depending on the inflammatory microenvironment, these cells are capable of mediating immunosuppression through secretion of antiinflammatory cytokines, recruitment of regulatory immune cells, exhaustion of pro-inflammatory cells through nutrient sequestering, and facilitation of cell polarization to antiinflammatory phenotypes (14). Previous studies have demonstrated the ability of MDSCs to prolong cardiac graft survival in a murine model, which was significantly reduced when MDSCs were depleted (47, 48), and accordingly, M-MDSCs were shown to promote organ acceptance through recruitment of regulatory T cells in clinical kidney transplantation (49). Furthermore, studies in islet and heart transplantation have demonstrated an association between MerTK function and M-MDSC–mediated transplant tolerance, mainly through their ability to recruit regulatory T cells (50). Our findings similarly suggest an upregulation of IL-10 after adoptive transfer of exogenous M-MDSCs that could be related to Tregs, thereby suggesting multifaceted signaling pathways secondary to MDSC-mediated resolution of IRI. Thus, results from this study raise the interesting prospect of the role of MDSCs in preventing MerTK cleavage for enhancing immunosuppression, increasing inflammation resolution, and alleviating IRI after LTx.

There are limitations in this study that should be considered. The use of the hilar ligation model, which is self-resolving, provides us a high-throughput way to assess the processes of resolution but does not recapitulate the entire clinical process of LTx, which includes cold preservation and donor-recipient characteristics that can further influence lung IRI. However, the use of an allogeneic LTx model circumvents these concerns, validating the observed findings. Second, the human translation of these experimental findings is currently limited because of the exclusion of standard clinical care immunosuppressive therapy in the preclinical murine models. We did not use immunosuppressant therapy in the LTx model, as our goal was to explore the inherent role of endogenous immune cell suppression by monocytic compartments without the influence of exogenous immunoregulation. However, since the standard clinical procedures involve immunosuppression in tissue after allograft, the broad extrapolation of the M-MDSC–mediated MerTK-driven immune regulation for a translational approach will require further exploration in relevantly designed future studies. Additionally, apart from MerTK, Tyro3 and Axl are a family of tyrosine kinase receptors that may influence the process of efferocytosis and resolution (51). These receptors may act individually or concomitantly in a disease-dependent setting and should be deciphered in subsequent studies of allograft injury (52). Also, the DEGs relative to efferocytosis in CLAD was not directly tested in a preclinical model of chronic rejection, as our murine models delineate PGD. However, since PGD is a primary contributor for CLAD development, analyzing efferocytosis-related gene patterns in patients with CLAD provides correlative insight into mechanisms contributing to chronic allograft rejection. Moreover, the single-cell RNA dataset utilizes healthy DT as the comparison group, which may not entail donor-recipient interactions and immunosuppressant-mediated changes that can contribute to alterations in gene expression. Therefore, to address these caveats, further investigation is required to delineate the role of impaired efferocytosis in sequential progression of PGD to CLAD in post-LTx cohorts for effective translational relevance of these preclinical findings and to decipher resolution of human lung transplant injury. In summary, our findings suggest a MerTK-mediated immunosuppressive mechanism for M-MDSCs in the resolution of lung IRI. The findings presented in this study characterize the untapped potential for the therapeutic use of M-MDSCs, as well as targeted compounds for preventing MerTK receptor cleavage, that can be enhanced ex vivo. As various cell death mechanisms have been proposed to mediate lung injury after transplant, the impending contribution of clearance pathways becomes of paramount importance to prevent graft rejection as well as enhance phagocytic capabilities to mitigate superimposed infections. Thus, future investigations should focus on enhancing the efferocytic ability of monocyte/macrophage populations through preservation of MerTK function for effective clinical translation in organ transplantation.

## Methods

Additional details are available in the Supplemental Methods.

### Sex as a biological variable.

Human LTx analysis included both men and women and no sex-dimorphic effects are reported. Our study examined male and female animals, and similar findings are reported for both sexes.

### Human BAL analysis.

BAL collection was performed on days 0 and 1 after LTx as routine surveillance bronchoscopy in accordance with the recommendations by the International Society for Heart and Lung Transplantation consensus statement for standardization of BAL in lung transplantation (53). Two 50 mL aliquots were instilled in the right middle lobe followed by aspiration. Samples were centrifuged at 500*g* for 5 minutes at 4°C, and supernatants were utilized in sol-MER analysis via ELISA, per manufacturer’s instructions (R&D Systems, Bio-Techne).

### Human single-cell RNA sequencing analysis.

We analyzed single-cell RNA-sequencing data of lungs explanted from 4 patients with CLAD and 3 normal donors (DT) from published dataset NCBI GEO GSE224210 for myeloid cell populations and subsequent differential gene expression (20). Sequencing analysis identified 12,061 genes, of which 1,940 were DEGs in CLAD versus DT in M-MDSCs (cluster 4) (Supplemental Table 1). Gene expression analysis was cross-referenced with efferocytosis-related and MerTK-related genes from GeneCard queries.

### Lung IRI model.

An established murine in vivo murine left lung hilar ligation model was used with 8- to 12-week-old male BALB/c (WT), C/EBPβ^–/–^ (*Cebpb*^–/–^), C57BL/6, *Mertk^–/–^* (The Jackson Laboratory), and MerTK-CR (gift from Bishuang Cai, Icahn School of Medicine at Mt. Sinai, New York, New York, USA) mice as previously described (42, 54). Briefly, animals were intubated, ventilated, and subjected to 1 hour of left lung ischemia via hilar occlusion. Reperfusion was initiated upon removal of hilar suture and commenced for 6 or 24 hours, and BAL as well as left lung tissue were harvested at the end of reperfusion durations. In separate groups, mice were treated with 5.0 × 10^6^ M-MDSCs via i.v. injection 24 hours before IRI. During both ischemia and reperfusion, mice were extubated and returned to their cage to minimize ventilator-induced injury.

### Brain-dead orthotopic lung transplantation.

Murine LTx was performed on brain-dead donors using C57BL/6 donor and BALB/c recipient mice for 24 or 72 hours of reperfusion, as previously described (55–57). Donors were subjected to brain death via slow inflation of a balloon catheter that was inserted via a paramedian borehole. Donor lungs were untouched for 3 hours in a period of “warm ischemia” followed by Perfadex flush through the main PA. Lungs were subsequently harvested and subjected to storage in 4°C Perfadex for 18 hours, then transplanted into recipient mice.

### Statistics.

Statistical evaluation was performed with GraphPad Prism 10 software. All values are presented as the mean ± standard error of the mean. One-way ANOVA followed by Tukey’s multiple-comparison test was performed to compare differences between 3 or more groups, as well as 2-tailed *t* test followed by Mann-Whitney *U* test. A value of *P* < 0.05 was considered statistically significant.

### Study approval.

Patients undergoing lung transplantation at University of Florida Health were consented in writing for BAL fluid collection before transplantation in accordance with the University of Florida IRB (IRB201900987). All murine studies were conducted with approval from the IACUC of the University of Florida under protocol 201810465.

### Data availability.

A Supporting Data Values file with all reported data values is available as part of the supplemental material, and other supporting material are available upon request to the corresponding author. Single-cell RNA-sequencing data are publicly accessible with NCBI’s GEO under GSE224210.

## Author contributions

AKS designed the study. VL, DJMK, ZT, MWS, and AKS performed experiments. VL, DJMK, ZT, JACV, MWS, CA, LLM, PAE, GRU, and AKS analyzed results. BS, MR, and AME provided human BAL specimens. GC and JACV performed single-cell sequencing analysis. VL and AKS prepared the manuscript with input from all authors.

## Supplementary Material

Supplemental data

Supplemental table 1

Supplemental table 2

Supplemental table 3

Supplemental table 4

Supporting data values

## Version 1 08/22/2024

In-Press Preview

## Version 2 10/08/2024

Electronic publication
