# Supplementary material for: MerTK-dependent efferocytosis by monocytic-MDSCs mediates resolution of ischemia/reperfusion injury after lung transplant
Source: JCI Insight. 2024 Aug 22;9(19):e179876. doi: 10.1172/jci.insight.179876 (PMC11466183; doi:10.1172/jci.insight.179876)
Supplement: Supplemental data [file jciinsight-9-179876-s210.pdf]

ONLINE DATA SUPPLEMENT

MerTK-dependent efferocytosis by monocytic-MDSCs mediates resolution of post-lung transplant ischemia-reperfusion injury

Victoria Leroy, Denny J. Manual Kollareth, Zhenxiao Tu, Jeff Arni C. Valisno, Makena Woolet-Stockton, Biplab Saha, Amir M. Emtiazjoo, Mindaugas Rackauskas, Lyle L. Moldawer, Philip A. Efron, Guoshuai Cai, Carl Atkinson, Gilbert R. Upchurch, Jr., Ashish K. Sharma

Supplemental Material:

1. **Supplemental Materials and Methods**
2. **References**
3. **Supplemental Figures**

Supplemental Figure S1. Dot plot of gene expression patterns utilized to identify Cluster 4 as M-MDSCs

Supplementary Figure S2. Gating strategy utilized to identify M-MDSCs ($CD45^+CD11b^+Ly6G^-Ly6C^+iNOS^+$) in lung tissue

Supplemental Figure S3. Administration of G-MDSCs does not attenuate lung IRI

Supplemental Figure S4. mRNA expression of MerTK in lung tissue

Supplemental Figure S5. *In vitro* generation of M-MDSCs

Supplemental Figure S6. M-MDSCs significantly abrogate immune cell activation

Supplemental Figure S7. Resolution of lung IRI is impaired in $MertK^{-/-}$ mice

Supplemental Figure S8. $Mertk^{-/-}$ and C57Bl/6 mice experience equivalent M-MDSC infiltration following 24hrs (IRI)

Supplemental Figure S9. Pro-inflammatory cytokine expressions are increased in $Mertk^{-/-}$ mice

Supplemental Figure S10. Enhanced neutrophil infiltration and activation occurs during failure of IRI resolution in $MertK^{-/-}$ mice

Supplemental Figure S11. MerTK-CR mice are protected following lung IRI

Supplemental Figure S12. MerTK-CR demonstrate decreased expression of pro-inflammatory cytokines

Supplemental Figure S13. MerTK-CR mice display decreased PMN infiltration and activation

Supplemental Figure S14. mRNA expression of MerTK in lung tissue of $Mertk^{-/-}$ and MerTK-CR mice

Supplemental Figure S15. WT and *Mertk*$^{-/-}$ M-MDSCs traffic equivalently to the lung after adoptive transfer administration

4. **Supplemental Tables**

Supplemental Table 1. Cluster 4 sequencing analysis

Supplemental Table 2. Efferocytosis-related genes

Supplemental Table 3. MerTK-efferocytosis related genes

Supplemental Table 4. Murine flow cytometry antibodies

SUPPLEMENTAL MATERIALS AND METHODS

Sex as a biological variable

Human LTx analysis included both male and female patients and no sex-dimorphic effects are reported. Our study examined male and female animals, and similar findings are reported for both sexes.

Human BAL analysis

BAL collection was performed on days 0 and 1 post-LTx as routine surveillance bronchoscopy in accordance with the recommendations by International Society for Heart and Lung Transplantation consensus statement for standardization of BAL in lung transplantation (1). Two 50mL aliquots were instilled in the right middle lobe followed by aspiration. Samples were centrifuged at 500g for 5 min at 4°C and supernatants were utilized in sol-MER analysis via ELISA, per manufacturer's instructions (R&D Systems, Minneapolis, MN).

Human single-cell RNA sequencing analysis

We analyzed scRNA-seq data of lungs explanted from four patients with chronic lung allograft dysfunction (CLAD) and three normal donors (DT). The original study has annotated data of 40 cell populations in endothelial, epithelial, lymphoid, myeloid and mesenchymal lineages (GSE224210)(2). In this study, we focused specifically on myeloid cell populations, which were filtered based on their original annotation, and reanalyzed cell clustering with the following steps: Data was analyzed using Seurat v5 after applying 'nFeature_RNA > 250 & nFeature_RNA < 4000 & percent.mt < 15' for quality control, 20,701 cells were included in further analysis (3). Data was normalized using the 'LogNormalize' method and CLAD and DT data were integrated using 30 identified anchors. Based on the top 20 principal components (PCs), the shared nearest neighbor (SNN) graph and Louvain algorithm were applied to identify 18 cell clusters, with a resolution parameter of 0.5. These cell clusters were visualized in a dimension-reduced space by the Uniform Manifold Approximation and Projection (UMAP) embeddings. We identified M-MDSC

cells based on the expression of *HLA-DRA, ITGAM, CD33, CD14, FUT4, IL-10, VEGFA* (4,5). Further, the CLAD data were compared to control data using Wilcoxon Rank Sum test and differentially expressed genes were detected by Log2FC>0.25, min.pct>0.01 and Bonferroni corrected p-value<0.05. Sequencing analysis identified 12,062 genes, of which 1,940 were differentially expressed genes (DEGs) in CLAD vs. DT in M-MDSCs (cluster 4) (Table 1). We cross-referenced these genes with a curated list of efferocytosis-related genes (ERG) generated from a GeneCards® and PubMed® query (efferocytosis; Table 2). We further refined our analysis by isolating differentially expressed (DE-) ERGs with a secondary GeneCards query, specifically targeting MerTK-related efferocytosis genes (MerTK; efferocytosis; Table 3) and cross-referenced with this list. This dual-step approach ensured a more precise identification of genes related to both MerTK and efferocytosis within the dataset.

Animal models of lung IRI

An established murine *in vivo* murine left lung hilar ligation model was used with 8-12 week old male BALB/c (wild-type; WT), C/EBPβ$^{-/-}$ (*Cebpb*$^{-/-}$), C57Bl/6, *Mertk*$^{-/-}$ (Jackson Laboratory), and MerTK-Cleavage Resistant (MerTK-CR; gift from Dr. Bishuang Cai, Icahn School of Medicine, Mt. Sinai, NY) mice as previously described (6,7). Drinking water and standard chow diet were provided *ad libitum*. All experiments were approved by and conducted in accordance to the Institutional Animal Care and Use Committee of the University of Florida (protocol#201810465). Briefly, mice were anesthetized with inhaled Pivetal® Isoflurane (Patterson Veterinary). Following anesthesia, mice were intubated with PE-60 tubing and connected to a pressure-controlled ventilator (Harvard Apparatus Co, South Natick, MA) for Mechanical ventilation with room air performed at 150 strokes/min, 0.5 cc stroke volume, and peak inspiratory pressure <20 cm H2O. Mice underwent thoracotomy followed by left hilar occlusion for 1hr (ischemia) then reperfusion for 6- or 24hrs. Reperfusion was achieved by removing the hilar suture. Sham animals were subjected to thoracotomy without hilar occlusion. To minimize ventilator-induced injury, mice were

extubated and returned to their cage during ischemic and reperfusion periods. Bronchoalveolar lavage (BAL) fluid and left lung tissue was harvested after the end of respective reperfusion time points for further analyses.

Additionally, a murine orthotopic lung transplant was used with brain dead donors using C57BL/6 donors and Balb/c recipient mice, as previously described (8-10). C57BL/6 donor mice were anesthetized by intraperitoneal injection of ketamine and xylazine (0.01 mg/g) and ventilated with a mixture of isoflurane and oxygen. Donors received a tracheotomy followed by insertion of a ventilation cannula for mechanical ventilation of 120/min respiration rate and a tidal volume of 300-400µL. A paramedian borehole was used to insert a 4F balloon catheter. The balloon was slowly inflated over a 10-minute period resulting in irreversible ischemia via brain stem compression. Donor lungs were left untouched for a 3-hour period of “warm ischemia” which was followed by a flush with 2mL of 4°C Perfadex® through the main pulmonary artery. Donor lungs were then harvested and stored for 18hrs in Perfadex® at 4°C prior to transplantation into recipient mice. Left lungs were transplanted utilizing cuff techniques into Balb/c recipient mice. Following left lung transplants, mice were sacrificed after 24- or 72hrs and BAL as well as lung tissue was harvested for further analyses.

Adoptive Transfer of M-MDSCs

In vitro generated M-MDSCs (detailed protocol outlined in a separate section below), were given to *separate groups* of mice at 5.0×10^6 M-MDSCs (derived from WT or *Mertk*$^{-/-}$ mice) via intravenous (i.v.) injection 24hrs prior to IRI. Separate experiments were conducted in which CellTrace Violet labeled WT or *Mertk*$^{-/-}$ M-MDSCs were administered to WT and/or *Cebpb*$^{-/-}$ mice. M-MDSCs were qualitatively assessed in lung tissue via microscopy and quantified in lung tissue via flow cytometry. For microscopy analysis, left lungs were harvest following IRI (24hrs), flash frozen in 2-methylbutane, and sectioned at 5µM using a Cryostat (Leica Biosystems). Images were acquired at 40x using a Nikon Eclipse Ti-U microscope (Nikon). For flow cytometry analysis, left lungs were harvested following IRI (6hrs) and IRI (24hrs) and subject to mechanical digestion

and red blood cell lysis to acquire a single cell suspension. Cells were stained with extracellular antibodies outlined in Supplemental Table 4. $CD45^+Ly6C^+Ly6G^-$CellTrace $Violet^+$ cells were analyzed via flow cytometry on a BD FACSymhony™A3 Cell Analyzer (BD Biosciences) and data was quantified using FlowJo Software.

Pulmonary function analysis

Pulmonary function was analyzed via a buffer-perfused, isolated mouse lung system (Hugo Sachs Elektronik), as previously described (11). After the reperfusion period, mice were anesthetized with ketamine and xylazine and a tracheostomy was performed. Ventilation of mice was provided with room air at 100 breaths/min at a tidal volume of 7µl/g body weight with a positive end expiratory pressure of 2 cm H_2O. The lungs were perfused at a constant flow of 60µl/g body weight/min with Krebs-Henseleit buffer (Sigma-Aldrich) containing 2% albumin 0.1% glucose and 0.3% HEPES (335–340 mOsmol/kg H_2O). The lungs were equilibrated on the system for 5-minutes before hemodynamic and pulmonary parameters were recorded for an additional 5 minutes by the PULMODYN data acquisition system (Hugo Sachs Elektronik).

Human bronchoalveolar lavage analysis

Patients undergoing lung transplantation at the University of Florida were consented for BAL collection with Institutional Review Board approval (#IRB201900987). BAL collection was performed as part of routine surveillance bronchoscopy by sequentially instilling two 50 mL aliquots in the right middle lobe followed by aspiration immediately after instilling the second aliquot using syringe suctioning, as recommended by International Society for Heart and Lung Transplantation consensus statement for standardization of BAL in lung transplantation (1). Samples were taken immediately post-transplantation (day 0; donor) and on post-operative day 1 in sterile tubes. BAL was normalized for return volume collected, centrifuged at 500*g* for 5 min at 4°C and supernatants were utilized for sol-Mer analysis.

Murine bronchoalveolar lavage analysis

Following lung function analysis, right lungs were ligated and left lungs were lavaged with 0.3mL of cold PBS. The return fluid was centrifuged at 500g for 5min at 4°C. The supernatant was collected and stored at –80°C for future analysis.

Cytokine and chemokine protein analysis

A mouse-specific Bio-Plex Cytokine Assay panel (Bio-Rad) was used to quantify cytokine and chemokine protein content in murine BAL fluid, as per the manufacturer's recommendations.

Myeloperoxidase measurement

Myeloperoxidase (MPO) was measured in murine BAL fluid using an MPO ELISA kit (R&D Systems), per the manufacturer's instructions.

Soluble MER Analysis

Human and murine BAL was analyzed for quantification of soluble MER using a sol-MER kit (R&D Systems) per the manufacturer's instructions.

M-MDSC quantification via Flow Cytometry

Left lungs of WT, *Cebpb*$^{-/-}$ and *Mertk*$^{-/-}$ mice subject to sham or IRI surgeries were harvested and subject to enzymatic dissociation via Lung Dissociation Kit and subsequent mechanical dissociation via GentleMACS per manufacturer's instructions (Miltenyi Biotec). The resulting cell suspension was filtered through a 70 μm cell strainer and then centrifuged at 300xg for 5 min at 4°C. Red blood cells were lysed with RBC Lysis Buffer (Roche, Millipore Sigma) by incubation for 2 min at 4°C. Following RBC lysis, cell suspensions were washed with PBS and centrifuged at 300xg for 5 min at 4°C. Cell pellets were resuspended in ice-cold PBS containing LIVE/DEAD™ Dye at 0.5μg per 1x10^6 cells for 30 minutes at 4°C. Cells were then washed twice in ice cold PBS, and blocked with 0.5μg per 1x10^6 cells mouse BD Fc Block™ (BD Biosciences) diluted in eBioscience™ Flow Cytometry Staining Buffer (Invitrogen). After 5 minutes of blocking antibody, samples were stained with a cocktail of fluorophore conjugated-antibodies as described in Supplemental Table 4 for 30 min at 4°C. Cells were then fixed using eBioscience Intracellular

Fixation and Permeabilization buffer set (eBioscience) per manufacturer's instructions. Cells were then subjected to intracellular staining of iNOS. Flow Cytometry was performed on a BD FACSymhony™ A3 Cell Analyzer and data was analyzed using FlowJo Software. M-MDSCs were identified as $CD45^{+}CD11b^{+}CD11c^{-}Ly6C^{+}Ly6G^{-}iNOS^{+}$ and G-MDSCs were identified as $CD45^{+}CD11b^{+}CD11c^{-}Ly6C^{lo}Ly6G^{+}$ as previously described (5,12) and quantified as a percentage of total $CD45^{+}$ cells.

Immunohistochemistry

Following IRI, left lungs were immediately harvested and incubated in 10% neutral buffered formalin fixation (Sigma-Aldrich) overnight. Tissues were embedded in paraffin and sectioned at 5μM. Neutrophil infiltration in lung tissue was performed by immunostaining lung sections with purified rat anti-mouse Ly-6G (Catalog No. 551459, BD Biosciences). Alkaline phosphatase–conjugated anti-rat immunoglobulin G (Catalog No. MP5404; Vector Laboratories) was used as the secondary antibody, and signals were detected with Vector® Red substrate kit (Vector Laboratories). Sections were counterstained with Mayer's Hematoxylin (Thermo Fisher Scientific). For each lung section, five random fields were captured at 20x. Neutrophil counts were acquired blindly via QuPath Software (13) and the counts were averaged per tissue sample.

In vitro generation of MDSCs

M-MDSCs were generated using *in vitro* methods as previously described (14). Briefly, cells were isolated from bone marrow of WT, C57BL/6, or *Mertk*$^{-/-}$ mice and cultured with granulocyte-macrophage stimulating factor (100ng or $2x10^{3}$ units per $3x10^{6}$ cells, Peprotech) in RPMI-1640 media containing 10% heat inactivated fetal bovine serum, 1% antibiotic-antimycotic, 1% L-glutamine, and 0.1% β-mercaptoethanol for 3 days. The resulting cell culture, consisting of M- and G-MDSCs, was further separated into Ly6G- and Ly6G+ subpopulations with Anti-Ly6G UltraPure Microbeads (Miltenyi Biotec) via AutoMACS magnetic based cell sorting. The Ly6G- population, representing M-MDSCs, was further cultured in the presence of LPS (0.1 ug/mL; Invitrogen) and IFN-γ (100 U/mL; Biolegend, San Diego, California) for 24hrs to induce

immunosuppression. Immunosuppressive M-MDSCs were subject to a Dead Cell Removal kit via magnetic separation (Miltenyi Biotec) and live M-MDSCs were then subsequently used for *in vivo* and *in vitro* experiments. G-MDSCs were isolated and purified similarly as outlined above. Following bone marrow cell culture, G-MDSCs were harvested from the heterogenous MDSC population utilizing Ly6G Isolation Kit (Miltenyi Biotec) via AutoMACS magnetic based cell sorting according to manufacture's instructions. This allowed for the purification of untouched (no bead-bound) G-MDSCs. These cells were then subject to Dead Cell Removal as described above and injected at $5x10^6$cells prior to IRI (6hrs).

In vivo efferocytosis

Efferocytosis was evaluated separately in WT mice following sham, 6hr- or 24hr-IRI. In endogenous efferocytosis evaluation, staurosporine-induced (1µM, Cayman Chemicals) apoptotic PMNs were labeled with pHrodo Red (Invitrogen) and administered at $1.0x10^6$ cells in 50µL of saline via intratracheal injection 1hr prior to ischemia. PMNs were acquired using a Neutrophil Isolation kit via AutoMACS (Miltenyi Biotec). Left lungs were harvested for efferocytosis analysis via flow cytometry. Endogenous M-MDSC efferocytosis was evaluated by identifying $CD45^+CD11b^+CD11c^-Ly6C^+Ly6G^-iNOS^+PE^+$ populations representing M-MDSCs with engulfed PE-labeled apoptotic neutrophils. In exogenous efferocytosis evaluation, adoptively transferred M-MDSCs were labeled with CellTrace™ Violet (Invitrogen) and given via i.v. 24hrs prior to injury. Left lungs were harvested following IRI. Efferocytosis by exogenously administered M-MDSCs was evaluated by identifying populations double positive for CellTrace™ Violet and PE (pHrodo Red). Efferocytosis percentage was calculated by dividing double positive populations by total M-MDSCs. Flow cytometry was performed on a BD FACSymhony™A3 Cell Analyzer and data was analyzed using FlowJo Software

In vitro efferocytosis

In vitro generated M-MDSCs from WT and *Mertk*$^{-/-}$ mice were co-cultured with live or staurosporine-induced apoptotic PMNs at a ratio of 1:3 for 6 hours and analyzed by flow

cytometry. M-MDSCs were labeled with CellTrace™ Violet and PMNs were labeled with CellTrace™ Yellow Dye prior to co-culture. Efferocytosis was evaluated via flow cytometry for double positive populations. Also, *in vitro* generated M-MDSCs were stained with PHK26 Red Fluorescent Cell Linker (Sigma-Aldrich) and plated at $0.2x10^6$ cells/well on collagen-gelatin coated culture chamber slides (Thermo Scientific) for 24 hours. PKH67 Green-labeled, staurosporine-induced apoptotic neutrophils were overlaid onto adherent M-MDSCs for 1hr. Chamber slides were washed with 1x PBS, fixed with 4% PFA, washed again with 1x PBS. Chamber slides were mounted to coverslips with Vectashield Vibrance mounting medium with DAPI (Vector Laboratories). Images were acquired at 40x using a Nikon Eclipse Ti-U microscope.

In vitro co-cultures

Primary invariant natural killer (iNKT) T cells (CD4+CD1d tetramer+ iNKT cells) were purified and cultured overnight in RPMI media containing 10% fetal bovine serum and 1% penicillin/streptomycin (Invitrogen), as previously described (15). iNKT cells were co-cultured with/without M-MDSCs ($0.25x10^6$ cells/well with 1:1 ratio) and exposed to normoxia or hypoxia–reoxygenation (HR) in 48-well culture dishes. A humidified, sealed hypoxic chamber (Billups-Rothenberg) was used to establish hypoxia exposure for 3hrs. Reoxygenation was achieved by removing the plates from the hypoxic chamber and placing them in a normoxic, humidified incubator (37°C, 5% CO_2, and 95% O_2) for an additional 3hrs. Cell culture supernatants were analyzed for IL-17 expression using ELISA, per the manufacturer's instructions (R&D systems).

RNA Extraction, cDNA generation, and real-time quantitative PCR

Total RNA was isolated from lung tissue and *in vitro* generated M-MDSCs using TRIzol according to the manufacturer's instructions. The concentration of total RNA was determined using NanoDrop™ (Thermo Fisher Scientific). iScript™ Reverse Transcription Supermix for RT-qPCR (Bio-Rad) was used to generate cDNA from 1000ng of total RAN. Subsequent detection and quantification of MerTK was performed using quantitative real-time PCR in a CFX96 Touch Real-Time PCR Detection System (Bio-Rad, Real-Time PCR Detection System (Bio-Rad,) using

SsoAdvanced™ Universal SYBR Green Supermix according to manufacturer's instructions. Assays were performed as follows: 1 cycle at 95°C for 2 min, then up to 40 cycles at 95°C for 05 sec and 63°C for 30 sec and then a melt curve was performed from 60°C to 95 °C (0.5 °C every 5 sec). For mRNA detection of MerTK, the following primers were used: MerTK (forward 5′-GCAAAAGTGACGTGTGGGCT-3′ and reverse 5′-GGGATCAGCACTCCAGCAAG -3′) and 18s (forward 5′-CGGCTACCACATCCAAGGAA -3′ and reverse 5′-AGCTGGAATTACCGCGGC-3′; Millipore Sigma). Results are reported as relative fold change and were calculated by utilizing the $2^{(-\Delta\Delta Ct)}$ method.

Statistical analysis

Statistical evaluation was performed with GraphPad Prism 10 software. All values are presented as the mean ± standard error of the mean (SEM). One-way ANOVA followed by Tukey's multiple comparison test was performed to compare differences between three or more groups, and T-test followed by Mann-Whitney test. A value of $P< 0.05$ was considered as statistically significant.

Study Approval

Patients undergoing lung transplantation at University of Florida Health were consented for BAL fluid collection prior to transplantation in accordance with the University of Florida Institutional Review Board (#IRB201900987). All murine studies were conducted with approval from the Institutional Animal Care and Use Committee of the University of Florida under protocol #201810465.

Data availability

A Supporting Data Values file with all reported data values is available as part of the supplemental material and other supporting material are available upon request to the corresponding author. Single-cell RNA sequencing data is publicly accessible with NIH's Gene Expression Omnibus under GSE224210.

REFERENCES

1. Martinu T, et al. International Society for Heart and Lung Transplantation consensus statement for the standardization of bronchoalveolar lavage in lung transplantation. *J Heart Lung Transplant*. Jul 15 2020;doi:10.1016/j.healun.2020.07.006

2. Khatri A, et al. JAK-STAT activation contributes to cytotoxic T cell-mediated basal cell death in human chronic lung allograft dysfunction. *JCI Insight*. Mar 22 2023;8(6)doi:10.1172/jci.insight.167082

3. Hao Y, et al. Dictionary learning for integrative, multimodal and scalable single-cell analysis. *Nat Biotechnol*. May 25 2023;doi:10.1038/s41587-023-01767-y

4. Song Q, et al. Dissecting intratumoral myeloid cell plasticity by single cell RNA-seq. *Cancer Med*. Jun 2019;8(6):3072-3085. doi:10.1002/cam4.2113

5. Bronte V, et al. Recommendations for myeloid-derived suppressor cell nomenclature and characterization standards. *Nature Communications*. 2016/07/06 2016;7(1):12150. doi:10.1038/ncomms12150

6. Cai J, et al. MicroRNA-206 antagomiR–enriched extracellular vesicles attenuate lung ischemia–reperfusion injury through CXCL1 regulation in alveolar epithelial cells. *J Heart Lung Transplant*. Dec 2020;39(12):1476-1490. doi:10.1016/j.healun.2020.09.012

7. Leroy V, et al. Resolution of post-lung transplant ischemia-reperfusion injury is modulated via Resolvin D1-FPR2 and Maresin 1-LGR6 signaling. *J Heart Lung Transplant*. May 2023;42(5):562-574. doi:10.1016/j.healun.2022.12.013

8. Atkinson C, et al. Donor Brain Death Exacerbates Complement-Dependent Ischemia/Reperfusion Injury in Transplanted Hearts. *Circulation*. 2013/03/26 2013;127(12):1290-1299. doi:10.1161/CIRCULATIONAHA.112.000784

9. Krupnick AS, et al. Orthotopic mouse lung transplantation as experimental methodology to study transplant and tumor biology. *Nat Protoc*. 2009;4(1):86-93. doi:10.1038/nprot.2008.218

10. Cheng Q, et al. Donor pretreatment with nebulized complement C3a receptor antagonist mitigates brain-death induced immunological injury post-lung transplant. *Am J Transplant*. Oct 2018;18(10):2417-2428. doi:10.1111/ajt.14717

11. Sharma AK, et al. Natural killer T cell-derived IL-17 mediates lung ischemia-reperfusion injury. *Am J Respir Crit Care Med*. Jun 01 2011;183(11):1539-49. doi:10.1164/rccm.201007-1173OC

12. Eckert I, et al. In Vitro Generation of Murine Myeloid-Derived Suppressor Cells, Analysis of Markers, Developmental Commitment, and Function. In: Brandau S, Dorhoi A, eds. *Myeloid-Derived Suppressor Cells*. Springer US; 2021:99-114.

13. Bankhead P, et al. QuPath: Open source software for digital pathology image analysis. *Scientific Reports*. 2017/12/04 2017;7(1):16878. doi:10.1038/s41598-017-17204-5

14. Eckert I, et al. In Vitro Generation of Murine Myeloid-Derived Suppressor Cells, Analysis of Markers, Developmental Commitment, and Function. *Methods Mol Biol*. 2021;2236:99-114. doi:10.1007/978-1-0716-1060-2_10

15. Sharma AK, et al. NOX2 Activation of Natural Killer T Cells Is Blocked by the Adenosine A2A Receptor to Inhibit Lung Ischemia-Reperfusion Injury. *Am J Respir Crit Care Med*. May 1 2016;193(9):988-99. doi:10.1164/rccm.201506-1253OC

Supplemental Figure S1

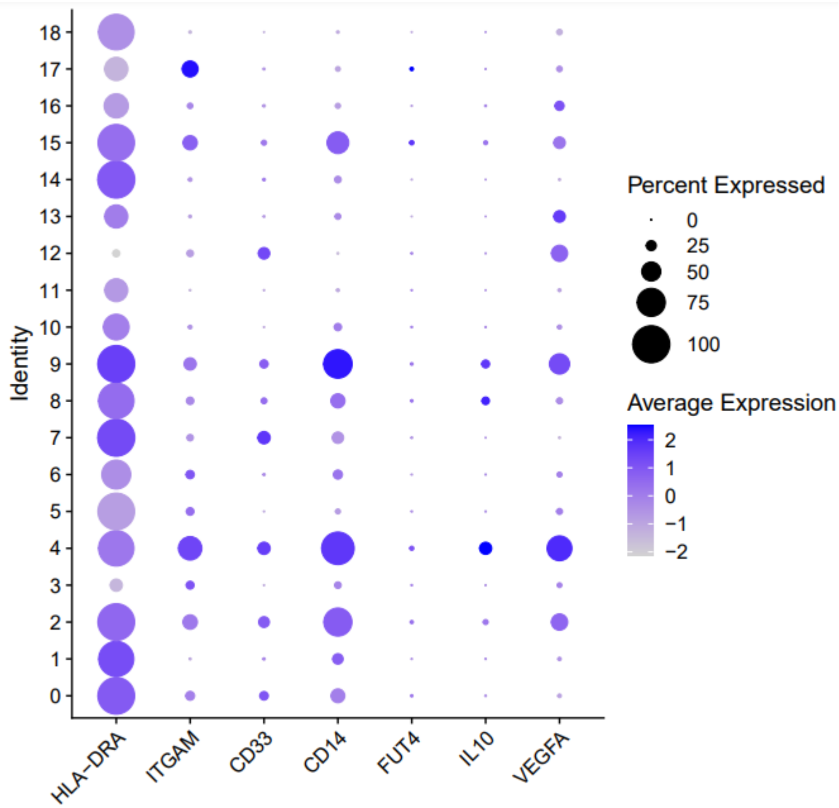

Supplemental Figure S1. Dot plot of gene expression patterns to identify Cluster 4 as M-MDSCs based on expression of *HLA-DRA, ITGAM, CD33, CD14, FUT4, IL-10,* and *VEGFA*.

Supplemental Figure S2

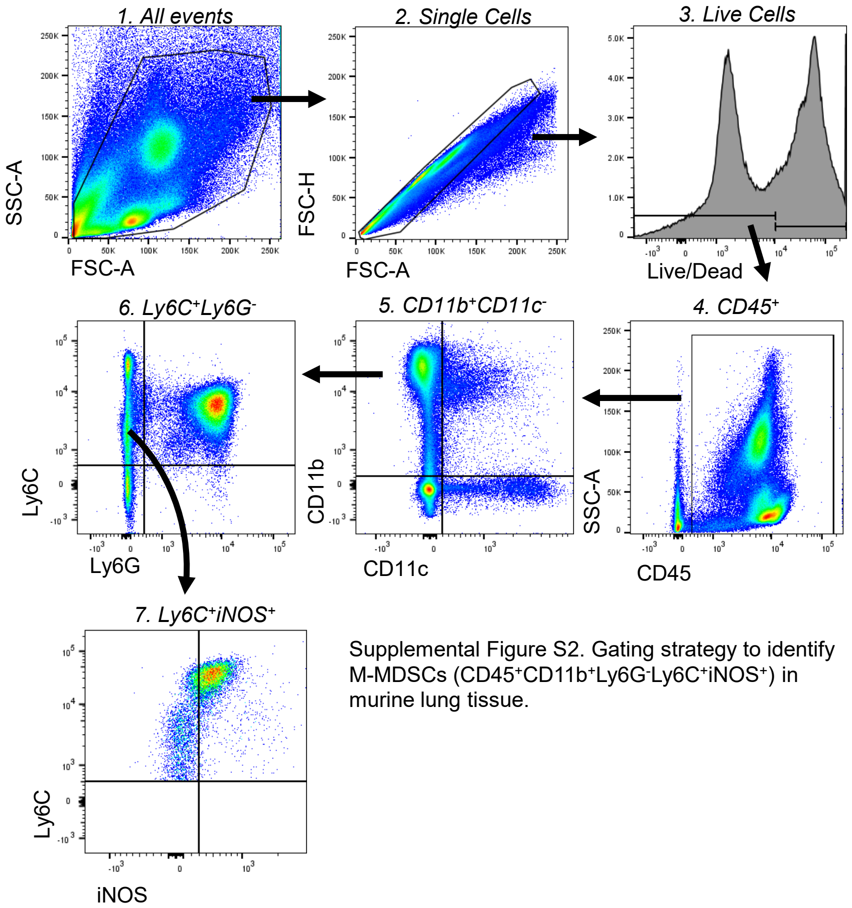

Supplemental Figure S2. Gating strategy to identify M-MDSCs ($CD45^{+}CD11b^{+}Ly6G^{-}Ly6C^{+}iNOS^{+}$) in murine lung tissue.

Supplemental Figure S3

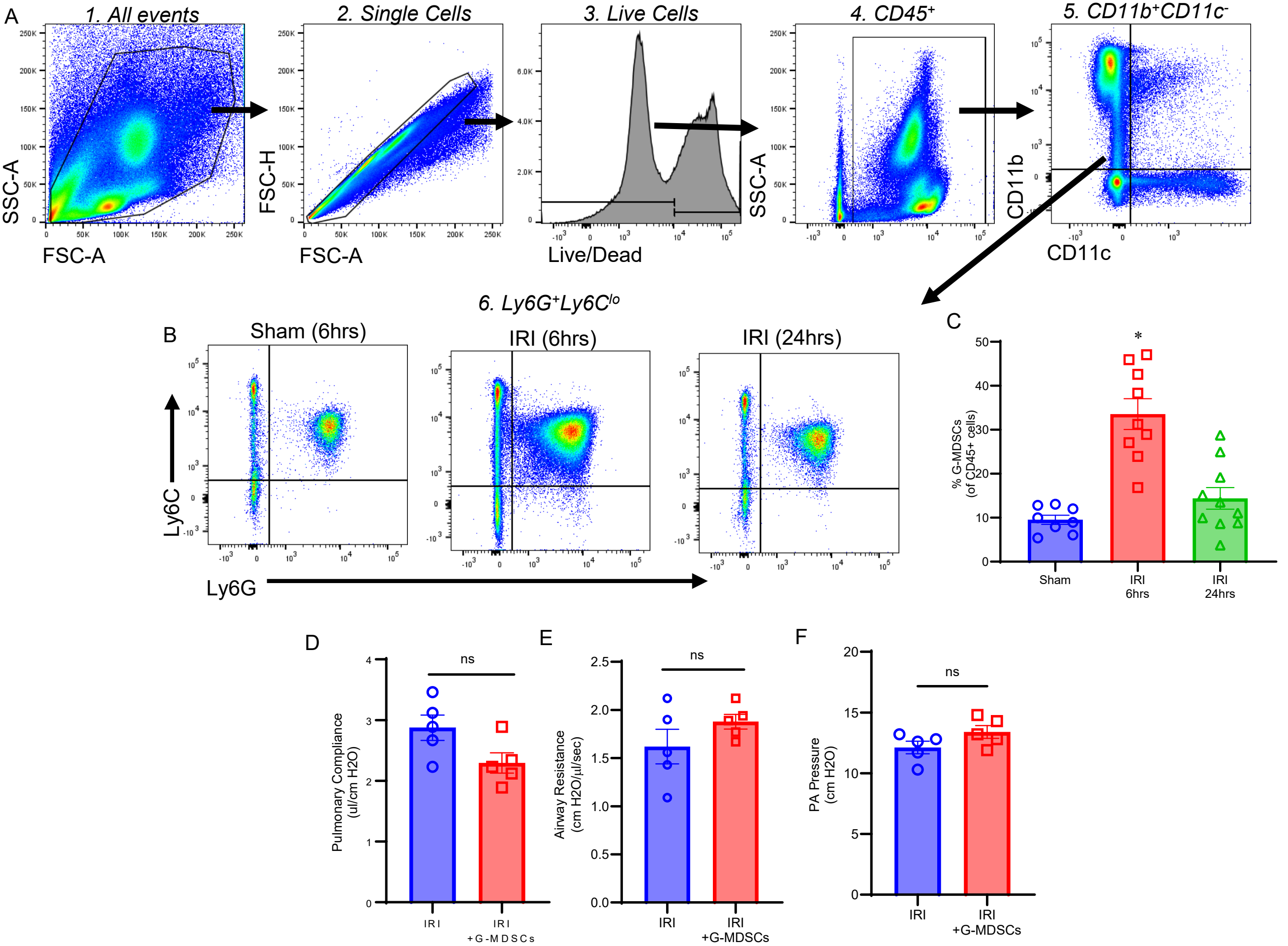

Supplemental Figure S3. G-MDSCs do not contribute to the resolution of lung IRI. **(A-C)** Quantification of G-MDSCs (CD45$^+$CD11b$^+$Ly6C^{lo}Ly6G$^+$) in lung tissue showed a significant increase following IRI (6hrs) compared to sham or IRI (24hrs) (*p<0.0001 vs. all other groups; *n*=8-10/group. Data analyzed by one-way ANOVA and Tukey's multiple comparisons test. **(D-F)** Administration of G-MDSCs does not attenuate lung IRI. Adoptive transfer of G-MDSCs to WT mice did not provide resolution of lung dysfunction after IRI (6hrs); ns, not significant; *n*=5/group. Data analyzed by Mann-Whitney test.

Supplemental Figure S4

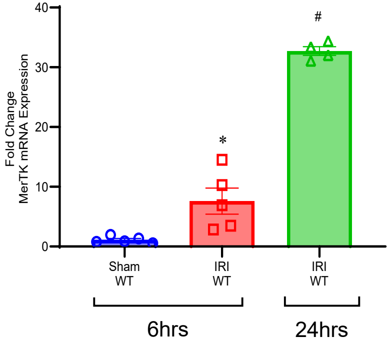

Supplemental Figure S4. A significant increase in MerTK mRNA expression was observed in lung tissue of WT mice during the resolution phase of IRI after 24hrs. *p<0.01 vs. sham; #p<0.0001 vs. IRI (6hrs); *n*=4-5/group. Data analyzed by one-way ANOVA and Tukey's multiple comparisons test.

Supplemental Figure S5

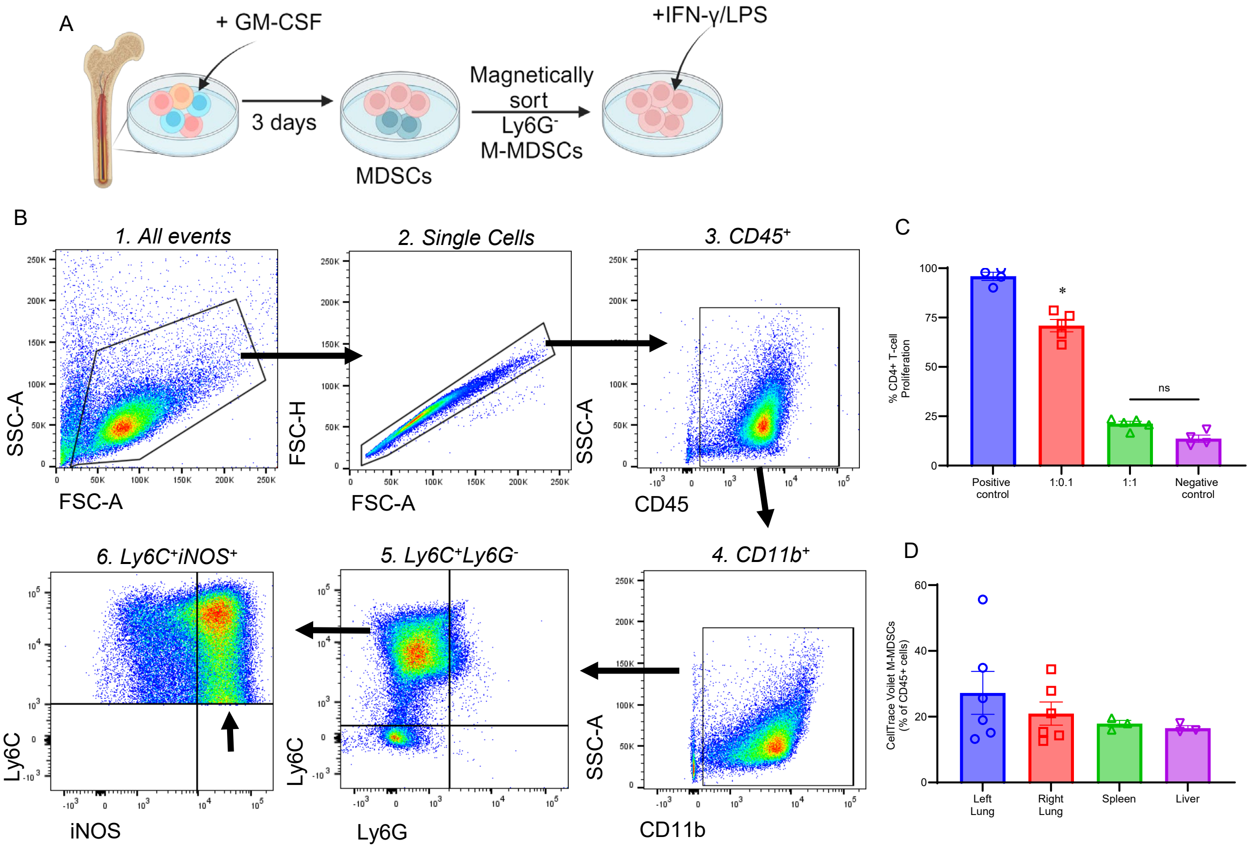

Supplemental Figure S5. In vitro generation of M-MDSCs. **(A)** Schematic depicting bone marrow harvest and in vitro culture to produce immunosuppressive M-MDSCs. **(B)** Representative gating strategy to confirm phenotype of M-MDSCs by $CD45^+CD11b^+Ly6G^-Ly6C^+iNOS^+$ cells. **(C)** Proliferation assays confirmed immunosuppressive ability of M-MDSCs to suppress $CD4^+$ T-cell proliferation in an M-MDSC dependent manner. Ratios are reported as T-cells:M-MDSCs. *$p<0.001$ vs. all other groups; ns, not significant; n=4-5/group. Data analyzed by one-way ANOVA and Tukey's multiple comparisons test. **(D)** Tracking of immunofluorescent labeled M-MDSCs confirm presence in the lung, spleen, and liver 24hrs after adoptive transfer administration. *n*=3-6/ group.

Supplemental Figure S6

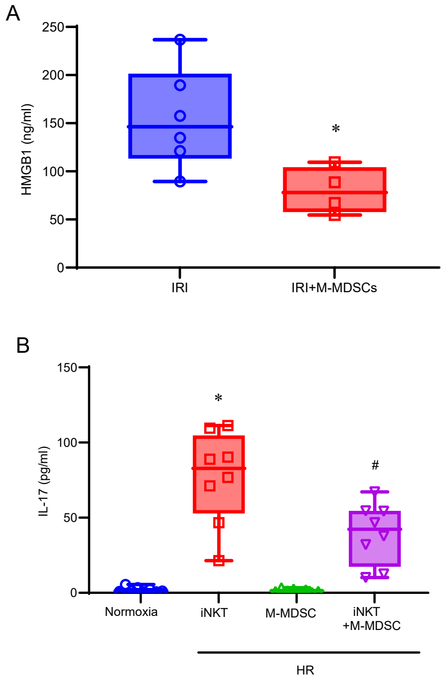

Supplemental Figure S6. M-MDSCs significantly abrogate immune cell activation. **(A)** Adoptive transfer of M-MDSCs from WT mice significantly attenuate HMGB1 expression in BAL of WT mice compared to untreated controls after IRI (6hrs). *p=0.01; n=4-6/group. Data analyzed by Mann-Whitney test. **(B)** *In vitro* hypoxia-reoxygenation (HR) upregulates iNKT cell-dependent IL-17 secretion compared to normoxic controls. Co-cultures of M-MDSCs with iNKT cells significantly decreases IL-17 expression in culture supernatants. *p<0.0001 vs. normoxia; #p=0.0019 vs. iNKT cells (HR); *n*=8/group. Data analyzed by one-way ANOVA and Tukey's multiple comparisons test.

Supplemental Figure S7

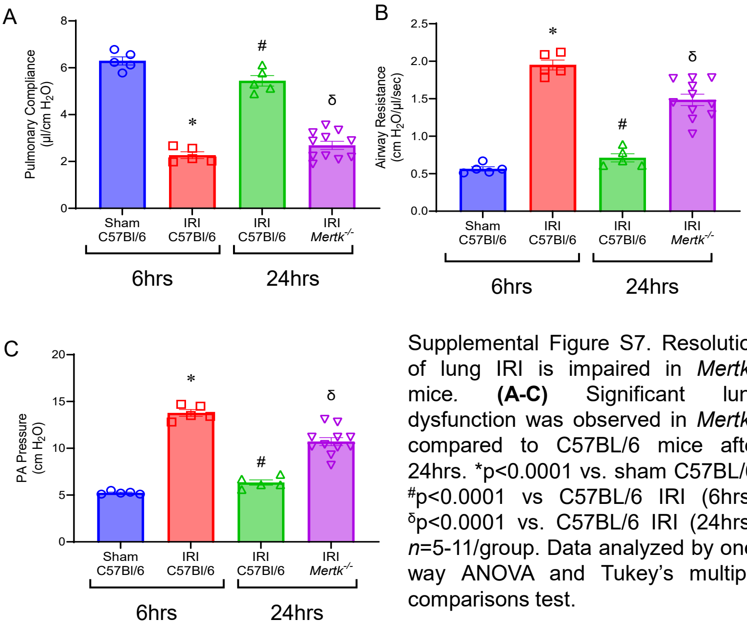

Supplemental Figure S7. Resolution of lung IRI is impaired in *Mertk*$^{-/-}$ mice. **(A-C)** Significant lung dysfunction was observed in *Mertk*$^{-/-}$ compared to C57BL/6 mice after 24hrs. *p<0.0001 vs. sham C57BL/6; $^{\#}$p<0.0001 vs C57BL/6 IRI (6hrs); $^{\delta}$p<0.0001 vs. C57BL/6 IRI (24hrs); *n*=5-11/group. Data analyzed by one-way ANOVA and Tukey's multiple comparisons test.

Supplemental Figure S8

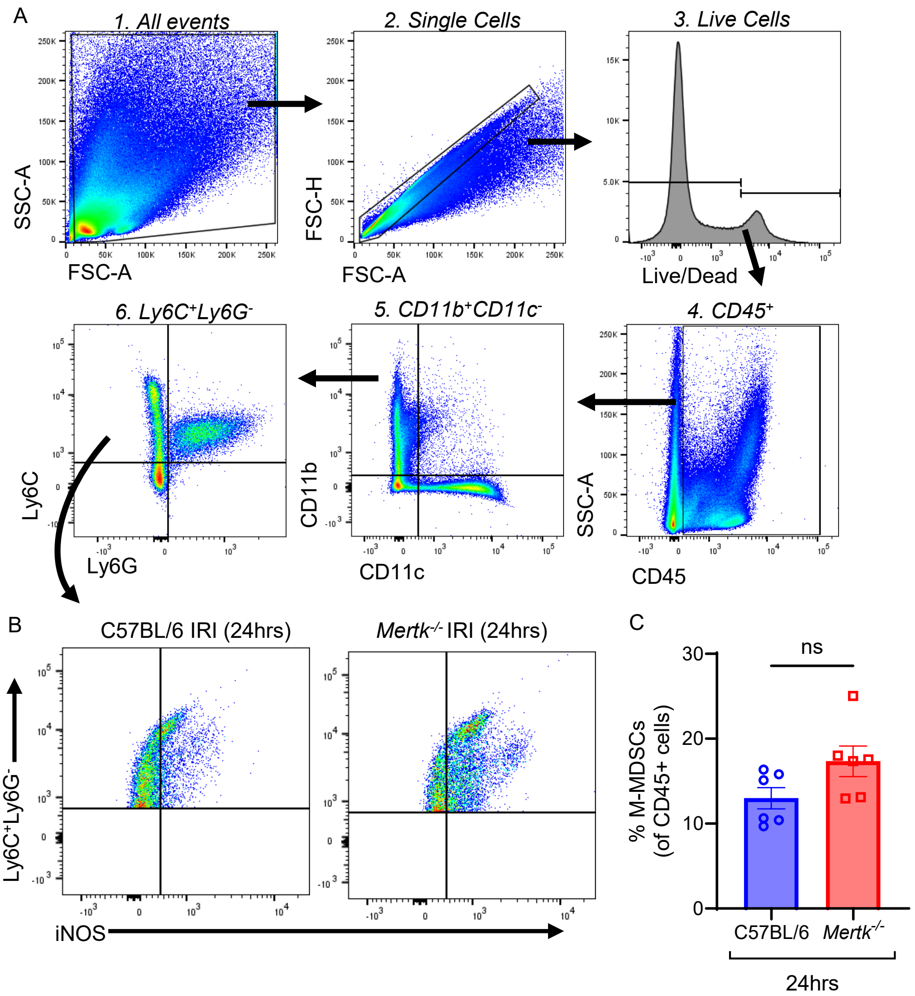

Supplemental Figure S8. *Mertk*$^{-/-}$ and C57/BL6 mice demonstrate no difference in M-MDSC infiltration following IRI (24hrs). **(A-B)** Flow cytometry gating strategy and representative plots for M-MDSC identification in lung tissue of *Mertk*$^{-/-}$ and C57/BL6 mice. **(C)** Quantification of M-MDSCs revealed no significant differences between *Mertk*$^{-/-}$ and C57/BL6 mice. ns, not significant; *n*=6/group. Data analyzed by Mann-Whitney test.

Supplemental Figure S9

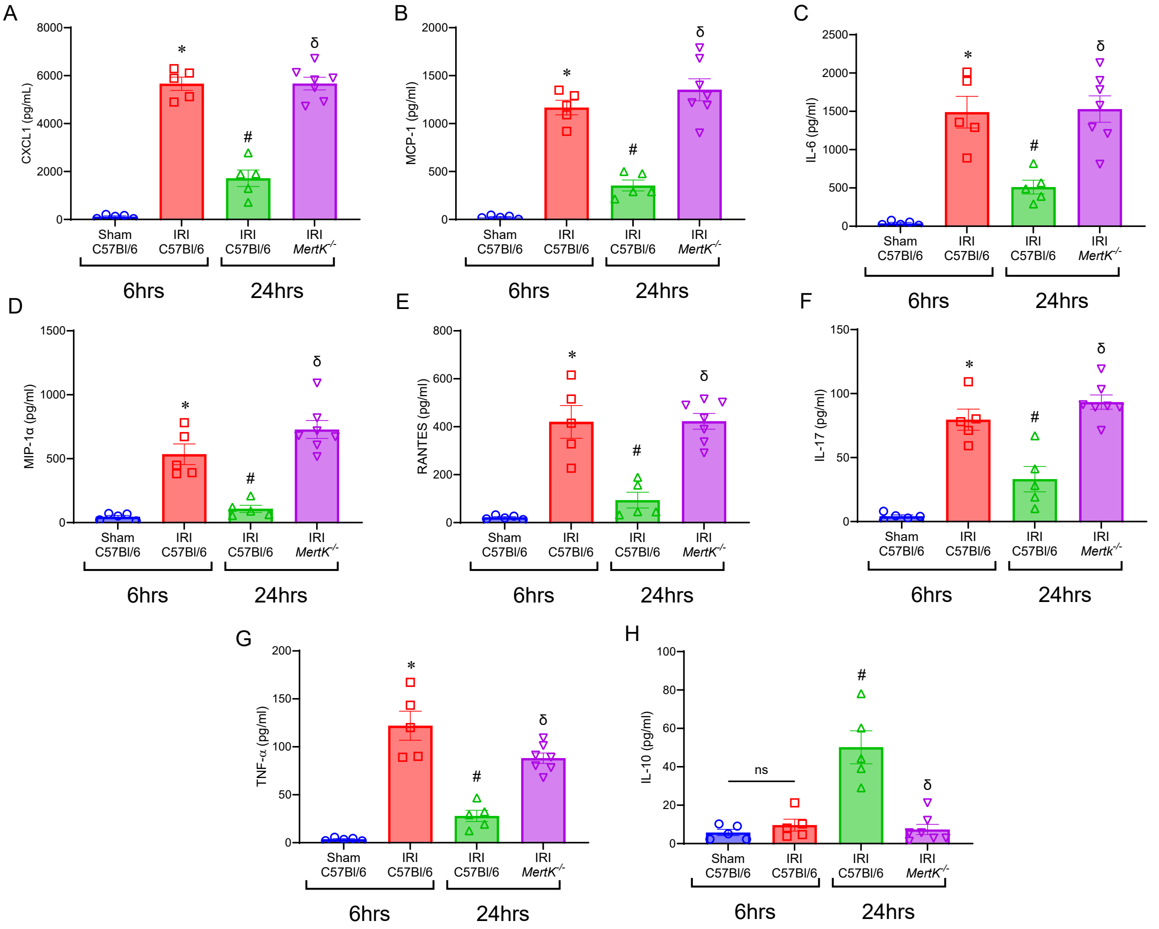

Supplemental Figure S9. Pro-inflammatory cytokine expressions are increased in *Mertk*$^{-/-}$ mice. **(A-H)** Pro-inflammatory cytokine expressions in BAL were significantly increased and anti-inflammatory IL-10 expression was significantly decreased in *Mertk*$^{-/-}$ compared to C57BL/6 mice after IRI (24hrs). *p<0.05 vs. sham C57BL/6; $^{\#}$p<0.05 vs C57BL/6 IRI (6hrs); $^{\delta}$p<0.05 vs. C57BL/6 IRI (24hrs); ns, not significant; *n*=5-7/group. Data analyzed by one-way ANOVA and Tukey's multiple comparisons test.

Supplemental Figure S10

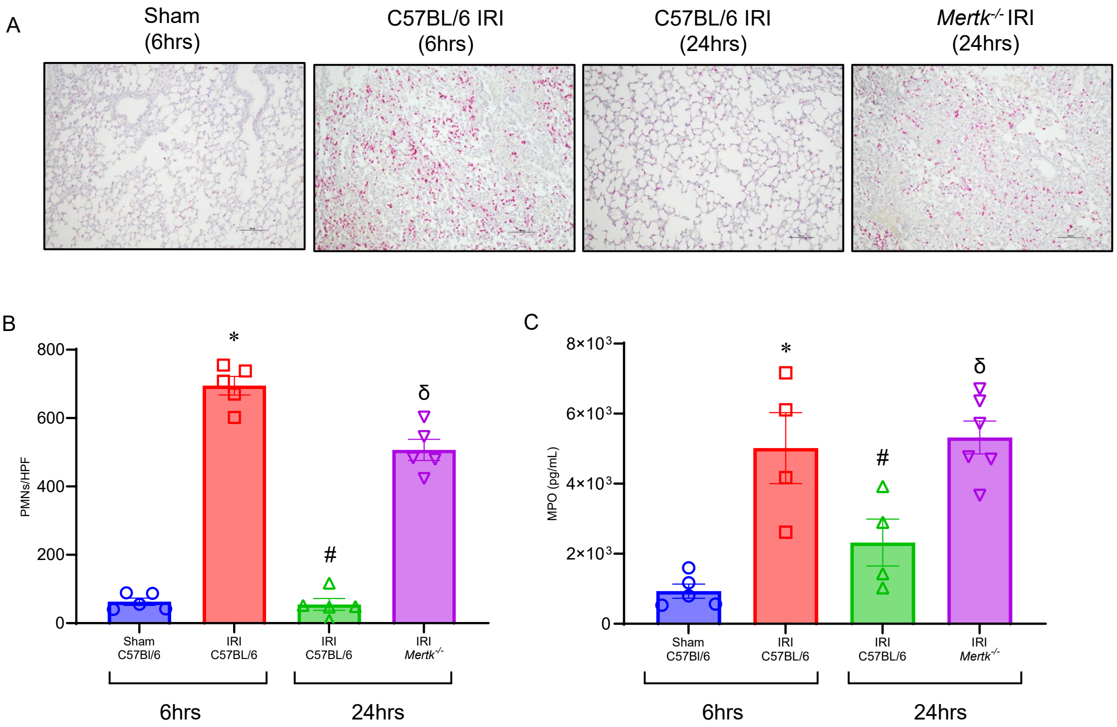

Supplemental Figure S10. Enhanced neutrophil infiltration and activation occurs during failure of IRI resolution in *Mertk*$^{-/-}$ mice. **(A-B)** Representative histological images and quantification of PMN infiltration demonstrate a significant increase in *Mertk*$^{-/-}$ mice compared to C57BL6 mice after 24hrs. *p<0.0001 vs. sham C57BL/6; $^{\#}$p<0.0001 vs. C57BL/6 IRI (6hrs); $^{\delta}$p<0.0001 vs. C57BL/6 IRI (24hrs); *n*=5/group. **(C)** MPO levels remain significantly increased in BAL from *Mertk*$^{-/-}$ mice compared to C57BL/6 mice after 24hrs. *p<0.001 vs. sham C57Bl/6; $^{\#}$p=0.04 vs C57BL/6 IRI (6hrs); $^{\delta}$p=0.01 vs. C57BL/6 IRI (24hrs); *n*=4-6/group. Data analyzed by one-way ANOVA and Tukey's multiple comparisons test.

Supplemental Figure S11

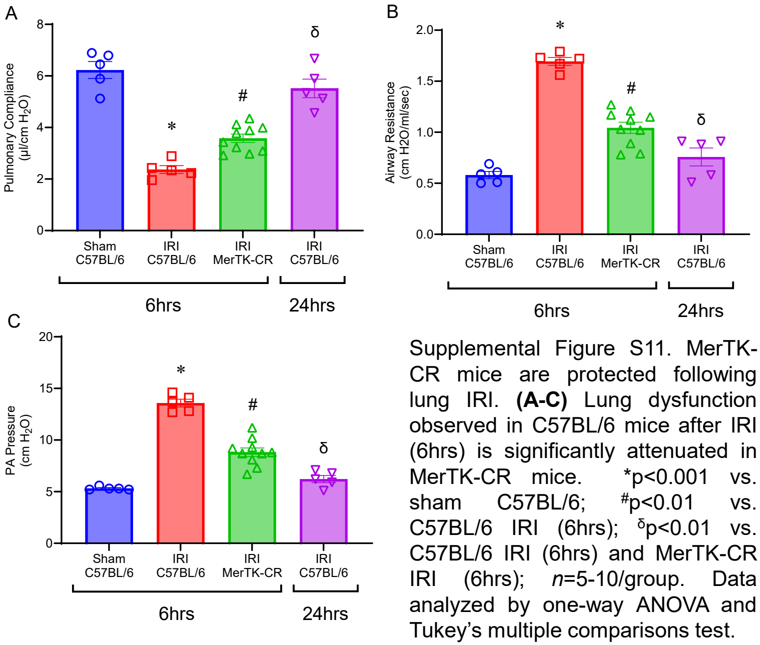

Supplemental Figure S11. MerTK-CR mice are protected following lung IRI. **(A-C)** Lung dysfunction observed in C57BL/6 mice after IRI (6hrs) is significantly attenuated in MerTK-CR mice. *$p<0.001$ vs. sham C57BL/6; #$p<0.01$ vs. C57BL/6 IRI (6hrs); δ$p<0.01$ vs. C57BL/6 IRI (6hrs) and MerTK-CR IRI (6hrs); *n*=5-10/group. Data analyzed by one-way ANOVA and Tukey's multiple comparisons test.

Supplemental Figure S12

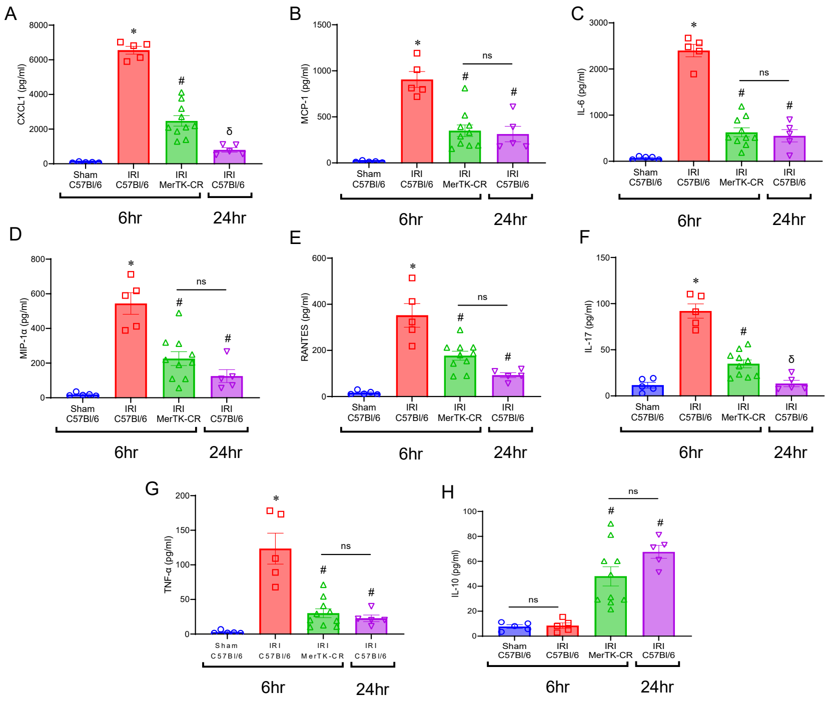

Supplemental Figure S12. MerTK-CR mice demonstrate decreased expression of pro-inflammatory cytokines. **(A-H)** MerTK-CR mice displayed significantly decreased pro-inflammatory cytokine and increased IL-10 expressions in BAL compared to C57BL/6 mice following IRI (6hrs). ns, not significant; *p<0.01 vs. sham C57BL/6; #p<0.02 vs. C57BL/6 IRI (6hrs); δp<0.02 vs. C57BL/6 IRI (6hrs) and MerTK-CR IRI (6hrs); *n*=5-10/group. Data analyzed by one-way ANOVA and Tukey's multiple comparisons test.

Supplemental Figure S13

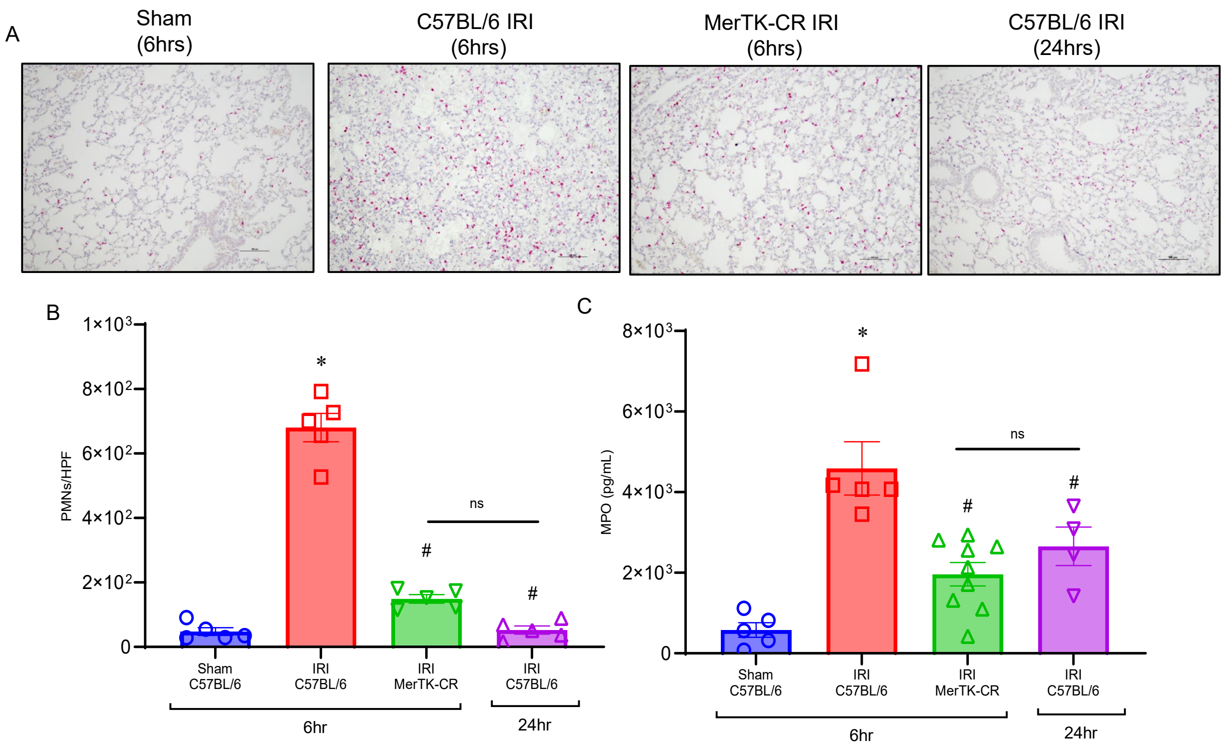

Supplemental Figure S13. MerTK-CR mice display decreased PMN infiltration and activation. **(A-B)** Histological staining for neutrophils and subsequent quantification revealed significant decrease in neutrophil infiltration in MerTK-CR mice compared to C57BL/6 mice following IRI (6hrs). *p<0.0001 vs. sham C57BL/6; $^{\#}$p<0.0001 vs. C57BL/6 IRI (6hrs); *n*=5/group. **(C)** MPO levels were also significantly decreased in BAL from MerTK-CR mice after 6hrs compared to C57BL/6. ns, not significant; *p<0.0001 vs. Sham C57BL/6; $^{\#}$p<0.001 vs C57BL/6 IRI (6hrs); *n*=4-9/group. Data analyzed by one-way ANOVA and Tukey's multiple comparisons test.

Supplemental Figure S14

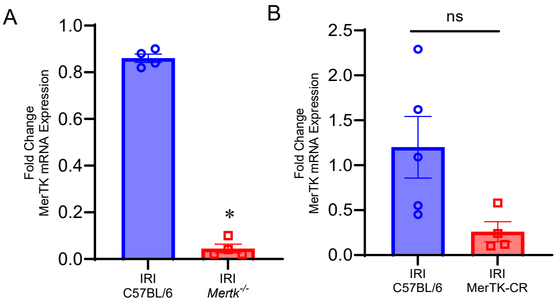

Supplemental Figure S14. mRNA expression of MerTK in lung tissue of *Mertk*$^{-/-}$ and MerTK-CR mice. **(A)** MerTK mRNA expression was significantly decreased in *Mertk*$^{-/-}$ mice compared to C57BL/6 mice following IRI (24hrs). **(B)** No significant differences were observed in MerTK mRNA expression in lung tissue of MerTK-CR mice compared to C57BL/6 mice after IRI (6hrs). ns, not significant; *p<0.0001; n=4-5/group. Data analyzed by Mann-Whitney test.

Supplemental Figure S15

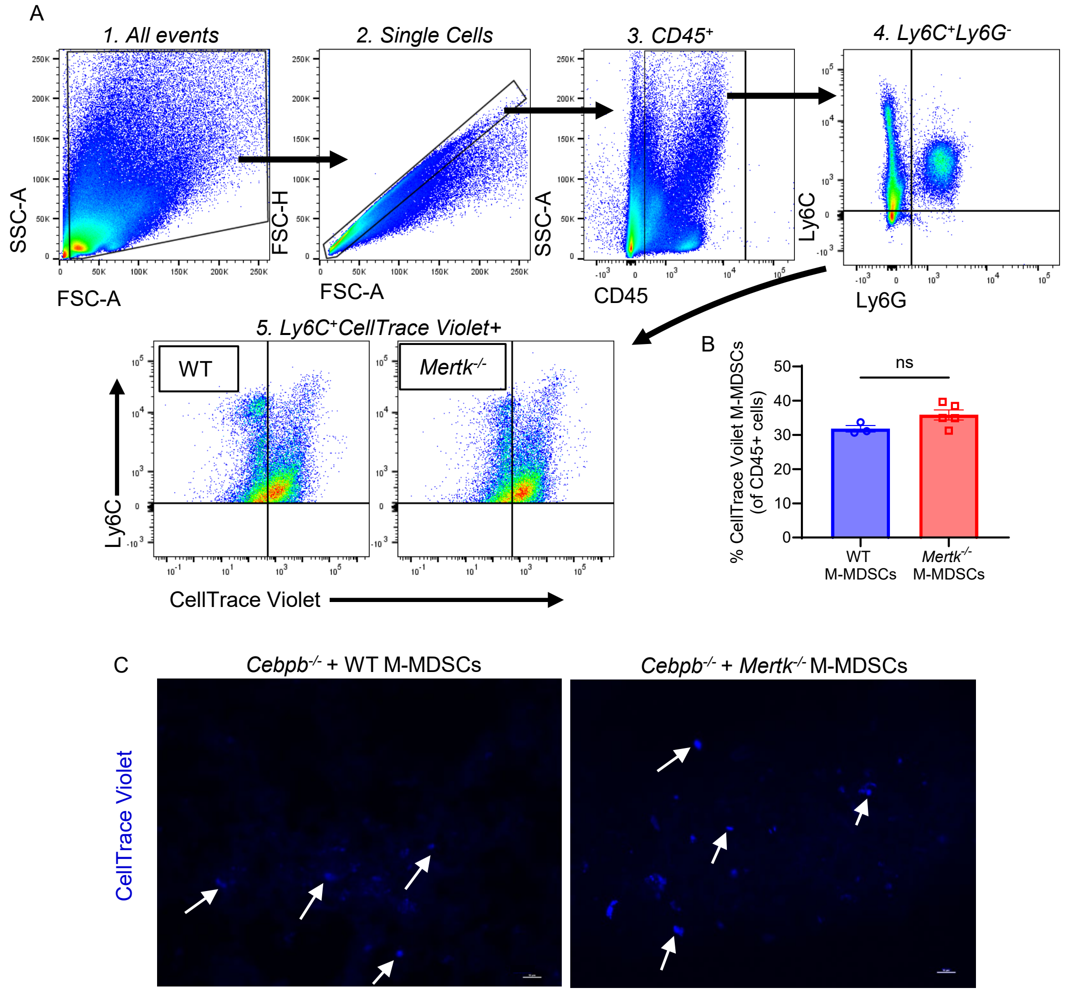

Supplemental Figure S15. M-MDSCs from WT and *Mertk*$^{-/-}$ traffic to the lung after adoptive transfers. **(A)** Representative flow cytometry plots identifying CellTrace Violet^{+} WT and *Mertk*$^{-/-}$ M-MDSCs in lung tissue of *Cebpb*$^{-/-}$ mice. **(B)** Quantification of adoptive transfer of CellTrace Violet^{+} M-MDSCs from WT and *Mertk*$^{-/-}$ mice to Cebpb-/- mice reveal no significant differences in the lung tissue after IRI (24hrs). ns, no significant; *n*=3-5/group. Data analyzed by Mann-Whitney test. **(C)** Representative immunofluorescence images confirm presence of CellTrace Violet^{+} WT and *Mertk*$^{-/-}$ M-MDSCs in lung tissue of *Cebpb*$^{-/-}$ mice. Scale bar is 10μM.
